# The Temperature-Dependent Effectiveness of Platinum-Based Drugs Mitomycin-C and 5-FU during Hyperthermic Intraperitoneal Chemotherapy (HIPEC) in Colorectal Cancer Cell Lines

**DOI:** 10.3390/cells9081775

**Published:** 2020-07-25

**Authors:** Roxan F.C.P.A. Helderman, Daan R. Löke, Jan Verhoeff, Hans M. Rodermond, Gregor G.W. van Bochove, Menno Boon, Sanne van Kesteren, Juan J. Garcia Vallejo, H. Petra Kok, Pieter J. Tanis, Nicolaas A.P. Franken, Johannes Crezee, Arlene L. Oei

**Affiliations:** 1 Laboratory for Experimental Oncology and Radiobiology (LEXOR), Center for Experimental and Molecular Medicine (CEMM), Amsterdam University Medical Centers (UMC), University of Amsterdam, Cancer Center Amsterdam, P.O. Box 22700, 1100 DE Amsterdam, The Netherlands; f.c.helderman@amsterdamumc.nl (R.F.C.P.A.H.); h.rodermond@amsterdamumc.nl (H.M.R.); g.g.vanbochove@amsterdamumc.nl (G.G.W.v.B.); mennoboon1@gmail.com (M.B.); sanne@vankesteren.net (S.v.K.); n.a.franken@amsterdamumc.nl (N.A.P.F.); 2 Department of Radiation Oncology, Amsterdam UMC, University of Amsterdam, P.O. Box 22700, 1100 DE Amsterdam, The Netherlands; d.r.loke@amsterdamumc.nl (D.R.L.); h.p.kok@amsterdamumc.nl (H.P.K.); h.crezee@amsterdamumc.nl (J.C.); 3 Department of Molecular Cell Biology & Immunology, Amsterdam Infection & Immunity Institute and Cancer Center Amsterdam, Amsterdam UMC, P.O. Box 7057, 1007 MB Amsterdam, The Netherlands; j.verhoeff@amsterdamumc.nl (J.V.); jj.garciavallejo@amsterdamumc.nl (J.J.G.V.); 4 Department for Surgery, Amsterdam UMC, University of Amsterdam, Cancer Center Amsterdam, P.O. Box 22700, 1100 DE Amsterdam, The Netherlands; p.j.tanis@amsterdamumc.nl

**Keywords:** hyperthermic intraperitoneal chemotherapy, HIPEC, colorectal cancer, platinum-based drugs, mitomycin-C, 5-fluorouracil, hyperthermia

## Abstract

Cytoreductive surgery (CRS) followed by hyperthermic intraperitoneal chemotherapy (HIPEC) is a treatment with curative intent for peritoneal metastasis of colorectal cancer (CRC). Currently, there is no standardized HIPEC protocol: choice of drug, perfusate temperature, and duration of treatment vary per institute. We investigated the temperature-dependent effectiveness of drugs often used in HIPEC. Methods: The effect of temperature on drug uptake, DNA damage, apoptosis, cell cycle distribution, and cell growth were assessed using the temperature-dependent IC50 and Thermal Enhancement Ratio (TER) values of the chemotherapeutic drugs cisplatin, oxaliplatin, carboplatin, mitomycin-C (MMC), and 5-fluorouracil (5-FU) on 2D and 3D CRC cell cultures at clinically relevant hyperthermic conditions (38–43 °C/60 min). Results: Hyperthermia alone decreased cell viability and clonogenicity of all cell lines. Treatment with platinum-based drugs and MMC resulted in G2-arrest. Platinum-based drugs display a temperature-dependent synergy with heat, with increased drug uptake, DNA damage, and apoptosis at elevated temperatures. Apoptotic levels increased after treatment with MMC or 5-FU, without a synergy with heat. Conclusion: Our in vitro results demonstrate that a 60-min exposure of platinum-based drugs and MMC are effective in treating 2D and 3D CRC cell cultures, where platinum-based drugs require hyperthermia (>41 °C) to augment effectivity, suggesting that they are, in principle, suitable for HIPEC.

## 1. Introduction

Colorectal cancer (CRC) is the third most common cancer worldwide, with 1.2 million new cases each year [1,2]. Metastases are the main cause of cancer-related deaths in most cancers, including CRC [3]. In 5–10% of patients diagnosed with CRC, peritoneal metastases (PMCRC) are already present and the incidence increases to 20–60% in recurrent disease [4,5]. PMCRC is associated with poor prognosis, averaging only 5–24 months and a 5-year survival rate between 0 and 22% [6,7].

For these patients, the only effective treatment with curative intent is cytoreductive surgery (CRS) to eradicate all macroscopic tumor, often combined with hyperthermic intraperitoneal chemotherapy (HIPEC). During HIPEC, the chemotherapeutic solution heated to 40–43 °C is circulated through the peritoneum for a period of 30 min, and up to 2 h [8,9].

Hyperthermic temperatures enhance cytotoxicity of the chemotherapy, resulting in increased killing of remaining microscopic tumors after CRS, reducing the risk of tumor recurrence [10,11]. The type of drug, drug concentrations, carrier solution, volume and temperature of the perfusate, duration of treatment, delivery technique, and patient selection all impact the efficacy of HIPEC [12]. Currently, there is no standardized HIPEC protocol for PMCRC, the choice of drug and temperature level are determined per institute [12].

The most commonly used drugs for HIPEC in PMCRC are oxaliplatin and mitomycin C (MMC) [12]. Common HIPEC regimes are 30 min with oxaliplatin at 42–43 °C or MMC at 41 °C for 90 min. The biological rationale for particular chemotherapeutic drugs, particular heating periods, and specific temperatures is currently lacking.

The most used agent MMC is an antibiotic chemotherapeutic agent isolated from Streptomyces caespitosus. MMC causes crosslinks in the DNA and thereby inhibits DNA synthesis [13]. MMC is used against a wide spectrum of tumors, especially gastrointestinal cancers. MMC is considered synergistic with heat and cell cycle non-specific and therefore suitable for application of HIPEC [13,14].

Oxaliplatin, the third generation platinum-based anticancer drug, contains a 1,2-diaminocyclohexane (DACH) ligand and oxalate as a leaving group [15], resulting in an increased uptake compared to first and second generation platinum-based drugs.

The first platinum anticancer drug cisplatin is widely used to treat various types of cancers including ovarian, testicular, bladder, colorectal, lung, and head and neck cancers [16,17]. Cisplatin crosslinks with purine bases on the DNA, interfering with DNA repair mechanisms, causing DNA damage and inducing apoptosis [16]. The second generation platinum-based drug carboplatin was developed to reduce the dose-limiting toxicity of cisplatin [15,16] with a reduced toxicity profile for carboplatin due to the bidentate cyclobutane dicarboxylate ligand [18].

Oxaliplatin was developed to overcome tumor resistance to cisplatin and carboplatin. Oxaliplatin mainly forms crosslinks on the adjacent guanine bases or between guanine and adenine; oxaliplatin-DNA adducts are more efficient in inhibiting DNA synthesis [16,19].

Oxaliplatin is used systemically to treat the majority of gastrointestinal tumors, often in combination with 5-fluorouracil (5-FU) [20]. 5-FU is a nucleoside metabolic inhibitor, minimally synergistic with heat, cell cycle-specific, and often used during early intraperitoneal chemotherapy (EPIC). 5-FU can also be administered intravenously before or after HIPEC. 5-FU acts during the S-phase of the cell cycle and inhibits DNA synthesis by blocking the thymidylate synthetase conversion of deoxyuridylic acid to thymidylic acid [21].

Tumor features also have a major impact on the outcome of HIPEC. Primary CRC can be classified based on gene expression profiling into four consensus molecular subtypes (CMS). These subtypes distinguish biological characteristics and prognostic significance [22]. For most patients suffering from PMCRC, the primary tumors can be classified as the mesenchymal subtype CMS4 [23]. CMS4 has the greatest propensity to form distant metastases [23]. CMS1 displays immune activation and is microsatellite instable with a relatively good prognosis [22]. CMS2 subtypes show upregulation of the WNT and MYC pathway signaling, whereas CMS3 is enriched with activation of metabolic pathways [24]. Currently, treatment protocols and drug selection do not take specific biological features of CRC and PMCRC into account. Patient selection based on the CMS classification can lead to a better and more personalized treatment. The choice of temperature, drug, and duration of the treatment could be adjusted based on the CMS subtype of the tumors, potentially leading to a better treatment outcome.

The aim of our study is to investigate the temperature-dependent effectiveness of the most frequently used chemotherapeutic agents for PMCRC. Detailed data are required to develop CMS-type and patient-specific HIPEC protocols. In this study, the efficacy of different drugs at different temperatures is investigated in several 2D and 3D in vitro CRC models. CMS1 and CMS4 subtype CRC cell lines were used to study the effects on cell viability, platinum uptake, DNA damage, apoptosis, cell cycle distribution, cell growth, and clonogenicity. The effect on cell viability was also studied in CMS4 subtype CRC and normal colon organoids. In addition, the influence of p53 protein function, which plays a central role in the regulation of the cell cycle after DNA damage, is studied [25]. The results of our study should give insights on which drug at which temperature might be used to optimize the application of HIPEC for PMCRC.

## 2. Materials and Methods

We evaluated, in vitro, the temperature-dependent effectiveness of oxaliplatin, MMC, cisplatin, carboplatin, and 5-FU. CRC cell lines (CMS1 and CMS4 subtypes) were used to study the effects on cell viability, platinum uptake, DNA damage, apoptosis, cell cycle distribution, cell growth, and clonogenicity. CMS4 subtype CRC and normal colon organoids were used to study the effect in a 3D cell culture model.

A schematic overview of the treatment steps and performed assays is shown in Figure S1. After cells were seeded and adhered to the bottom of the plate, chemotherapeutic agents were added to the medium and plates were incubated in a thermostatically regulated water bath to perform hyperthermia treatment. After a 60-min in vitro HIPEC treatment, the medium was refreshed and cells were placed in the incubator for the desired time of the experiment.

### 2.1. 2D and 3D Cell Cultures

CMS1 colorectal cancer (CRC) cells: RKO, HCT116, RC10.1, RC10.2, and RKO p53−/− are microsatellite instable. RC10.1, RC10.2, and RKO p53−/− are mutated RKO cells with decreased p53 levels and are used to study the role of p53 in in vitro HIPEC treatment. CMS4 CRC cells: MDST8, COLO320, and HUTU80 are microsatellite stable. The characteristics and pathway mutations of these cells are summarized in Figure S2A–D.

RKO, RC10.1, RC10.2, and RKO p53−/− cells were kindly provided by Dr. Kathleen Cho, University of Michigan (Ann Arbor, MI, USA). RKO cells with wild-type p53 were transfected with HPV16-E6-1 (RC10.1) and HPV16-E6-2 (RC10.2), resulting in a decrease of p53 levels and function. For selection, geneticin (200 µg/mL) was added to the complete medium of RC10.1 and RC10.2 cells to maintain the transfectants. Prior to treatment, cells were cultured in complete medium without geneticin for 3 days to minimize the influence of geneticin on the outcome of treatment. Wild-type RKO cells with a substitution of alanine for valine at codon 143 (mp53.13), resulted in a dominant negative p53 mutant.

HCT116, HUTU80, MDST8, COLO320, and T24 were all obtained from American Type Culture Collection (ATCC), RT112 was obtained from Sigma-Aldrich (Sigma Aldrich, Merck, KGaA, Darmstadt, Germany) and AG1522 was obtained from the Coriell Institute.

RKO, RC10.1, RC10.2, RKO p53−/−, and T24 were cultured in McCoy’s 5a medium (Gibco, Rockville, MD, USA); HCT116 and HUTU80 in DMEM/F12 medium (Gibco); MDST8 and COLO320 in RPMI medium (Gibco); and RT112 and AG1522 in EMEM medium (Lonza, Allendale, NJ, USA). All media contained 25 mM HEPES, supplemented with 10% fetal bovine serum (Gibco) and 1% penicillin/streptomycin/glutamine (Gibco). Cells were maintained at 37 °C in a humidified atmosphere of 5% CO_2_ in air.

Organoids which are assumed to represent the in vivo PMCRC better than 2D cell cultures, were isolated from different patients with colorectal cancer in accordance with the rules of the medical ethical committee of the Amsterdam UMC and University of Palermo. CO147 was established in the Amsterdam UMC, Amsterdam. RC511 was originated from Palermo, Italy. Colon-wild type (colon-WT) was isolated from a healthy part of the colon in the Amsterdam UMC, Amsterdam. The characteristics and pathway mutations of these organoid models are summarized in Figure S2C,D.

CO147, RC551, and colon-WT were cultured as organoids in matrigel matrix (Corning, New York, NY, USA). All organoids were cultured in advanced DMEM/F12 (Gibco) medium supplemented with 50× diluted N2 supplement (Life Technologies, Bleiswijk, the Netherlands), 50 µM hepes (Life Technologies), 2 mM GlutaMAX-I (Invitrogen, Eugene, OR, USA) 10 µg/mL penicillin/streptomycin/glutamine (Gibco), and 10 µg/mL gentamycin (Lonza). The following growth factors were used: 250 ηg/mL Amphotericin B (Lonza), 1× B27 supplement (Life Technology), 1 mM N-Acetylcysteine (Sigma-Aldrich Merck), 5 mM [Leu15]-gastrin (Sigma-Aldrich, Merck), 10 mM Nicotinamide (Sigma-Aldrich Merck), 500 ηM Alk4/5/7/inhibitor (A83-01, Tocris Bioscience, Ellisville, MO, USA), 10 µM SB202190 (Sigma-Aldrich Merck), 125 ηg/mL EGF human recombinant (TEBU-BIO, Heerhugowaard, the Netherlands), 10 ηM Prostaglandin E2 (Santa Cruz, CA, USA), R-spondin conditioned medium, and Noggin conditioned medium. Culture medium specific for CO147 and RC511 was additionally supplemented with 10 µM Rock inhibitor/Y-26632 (Sigma-Aldrich Merck) and medium specific for colon-WT was additionally supplemented with WNT3A conditioned medium. Organoids were maintained at 37 °C in a humidified atmosphere of 5% CO_2_ in air.

### 2.2. In Vitro HIPEC Treatment

All cell lines were exposed to various concentrations of chemotherapeutic agents under hyperthermic conditions at clinically relevant temperatures: 38–43 °C for 60 min (plus an additional 10-min pre-heating) or 37 °C as a control. Stock-solutions of agents: cisplatin (Platosin, Pharmachemie, Haarlem, the Netherlands), carboplatin (Bio-connect, Huissen, the Netherlands), oxaliplatin (Cayman Chemical Company, Ann Arbor, MI, USA), mitomycin C (Bio-connect), and 5-FU (Teva Pharmaceuticals, Wilrijk, Belgium) were stored at 4 °C. Appropriate dilutions were freshly prepared for each experiment. Hyperthermia was performed by placing the plates in a thermostatically regulated water bath supplemented with 5% CO_2_. After treatment, medium was replaced with fresh medium (without chemotherapy) and cells were incubated at 37 °C in an atmosphere of 5% CO_2_ in air for the desired time of the specific assay.

### 2.3. Cell Viability Assay

Cell viability was assessed by PrestoBlue^®^ Cell Viability assays (Invitrogen). Cells were harvested and counted using an automated Luna cell counter (Logos Biosystems, Annandale, VA, USA) and 25,000 cells were plated in each well of a 48-wells plate (Greiner BioOne GmbH, Kremsmuenter, Austria). Plates were incubated overnight at 37 °C and subjected to in vitro HIPEC-treatment. After 2 days of incubation, the medium was replaced by 10-time diluted PrestoBlue^®^ Cell Viability Reagent in complete medium and incubated for 4 h at 37 °C. The extinction of every well was measured using a plate reader (Biotek synergy HTX) at 570 nm. Cell viability fractions were calculated by correcting the extinction of treated cells by the extinction of control cells. The drug concentrations required to eliminate 50 percent of the cancer cells (IC50) at 43 °C was used in further assays.

### 2.4. Nicoletti Assay

Cell cycle distribution and apoptosis was determined by the Nicoletti assay [26]. Cells were subjected to in vitro HIPEC-treatment, as described above. After treatment, medium was replaced with fresh medium (without chemotherapy) and cells were incubated at 37 °C for 48 h. Subsequently, cell pellets were collected and resuspended in Nicoletti buffer. Apoptosis levels were measured by flow cytometry (FACS Canto, BD Biosciences, San Jose, CA, USA) and analyzed with FlowJo v.10.2.

### 2.5. BrdU Staining

The effect of the treatment on cell cycle distribution was performed using thymidine analogue 5-Bromo-2′deoxy-uridine (BrdU, Sigma Aldrich, Merck, KGaA, Darmstadt, Germany), as described earlier [27]. Cells were subjected to in vitro HIPEC-treatment, as described above. Samples were analyzed using flow cytometry.

### 2.6. Cell Growth Assay

To study cell growth after different treatments, live imaging analysis was performed with the IncuCyte C3 (Sartorius, Goettingen, Germany). Cells were plated in appropriate density in 24-well plates (Greiner), incubated overnight at 37 °C and treated as described above. After treatment, the medium was refreshed and cells were incubated for 3 days in the IncuCyte at 37 °C in an atmosphere of 5% CO_2_ in air. Every 2 h, 4 pictures per well were taken. The phase area (confluence) of every well was calculated with the IncuCyte S3 software.

### 2.7. Cellular Platinum Uptake

The cellular uptake of platinum-based chemotherapeutic agents (cisplatin, oxaliplatin, and carboplatin) after hyperthermia treatment was assessed using cytometry by time of flight (CyTOF). Cells were subjected to in vitro HIPEC-treatment, as described above. After treatment, the medium containing the chemotherapeutic agent was replaced with complete fresh medium and cells were incubated at 37 °C for one hour. Subsequently, cells were collected and fixed for 1 h with 1.6% paraformaldehyde at 37 °C.

After fixation, cells were barcoded with a unique combination of palladium isotopes, according to the manufacturer’s protocol (Cell-ID™ 20-Plex Pd Barcoding Kit, Fluidigm, CA, USA). Pooled samples were stained for DNA using 125 nM iridium intercalator (Fluidigm) in Fix and Perm Buffer (Fluidigm) for 1 h at 37 °C. Before analysis on CyTOF-Helios, samples were washed twice with Cell Stain Buffer (CSB, Fluidigm) and twice with ddH2O. Events were recorded on CyTOF using standard tuning and QC settings in software version 6.7 at an event rate of 500 events per second. Data were normalized using bead standards according to the manufacturer’s protocol. The resulting FCS files were manually gated using Cytobank [28]. Singlets were selected using Gaussian parameters (Fluidigm). Single cell platinum uptake was assessed by measuring on Channel 194, which collects the signal emitted by the most abundant natural occurring isotope of platinum.

### 2.8. Clonogenic Survival Assay

To assess long-term cell survival of treated and untreated cells, clonogenic survival assays were performed as described before [29]. Cells were plated in appropriate density in 6-well plates (Greiner). Plates were incubated overnight at 37 °C and treated as described above. At 10 days after treatment, colonies were fixed by 6% glutaraldehyde and stained with 0.05% crystal violet. Surviving fractions were calculated by correcting the plating efficiency of treated cells with the plating efficiency of control cells.

### 2.9. γ-H2AX Staining

The induction of DNA double strand breaks (DSBs) after treatment was assessed by performing immunohistochemical stainings for γ-H2AX, as described before [30]. Cells were seeded on sterile cover slips (21 × 26 mm), placed in 60-mm cell culture dishes, and incubated overnight at 37 °C. Cells were treated as described above with oxaliplatin or MMC, in combination with or without hyperthermia at 42 °C for 1 h.

Digital image analysis was performed to determine the induction of γ-H2AX foci after treatment. Fluorescent pictures of γ-H2AX foci were obtained using Leica LAS-X software (Leica, Wetzlar, Germany). Slices of 61 stacks with a 30-µm interval between the slices along the *z*-axis of at least 50 cells were obtained using Leica DM6 microscope equipped with a CCD camera.

### 2.10. Statistical Analysis

Comparison of independent treatment groups for platinum uptake, number of foci, apoptosis, and survival factions were calculated using Mann–Whitney–U, a non-parametric test. Statistical analyses were performed using Prism version 8 (GraphPad Software, Inc., CA, San Diego, USA). Significant *p*-values are presented as: * *p* ≤ 0.05, ** *p* ≤ 0.01, *** *p* ≤ 0.001. All *p*-values are presented in Figure S11.

## 3. Results

### 3.1. Human Colorectal Cancer Cells Are Sensitive to Hyperthermia

**Figure 1:**
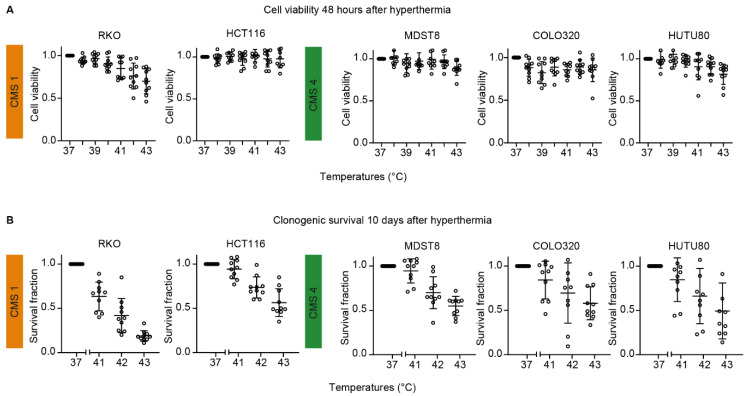
Human colorectal cancer cells are sensitive to hyperthermia. Five different human colorectal cancer cell lines: RKO, HCT116, MDST8, COLO320, and HUTU80, were used to study the sensitivity to hyperthermia. (**A**) Cell viability assays were performed at 48 h after hyperthermia treatment at 38, 39, 40, 41, 42, and 43 °C. (**B**) Long-term cell survival after hyperthermia treatment at 41, 42, and 43 °C was assessed using clonogenic survival assays performed at 10 days after treatment. For both experimental setups, cells at 37 °C represent the untreated controls. The mean of 10 independent experiments are presented.

The cell viability of RKO cells decreases significantly after hyperthermia treatment with a 30% reduction after a 43 °C treatment compared to control, whereas HCT116 does not show any differences in cell viability after hyperthermia treatment (Figure 1A). All CMS4 cell lines demonstrate decreased cell viability with almost 20% less cell viability of HUTU80 cells after treatment at 43 °C compared with untreated cells.

Long-term survival after hyperthermia treatment was also assessed. In Figure 1B, the results of clonogenic survival assays performed at 10 days after hyperthermia treatment at 41, 42, or 43 °C are shown. We do not show results for temperatures lower than 41 °C, as these did not result in decreased cell viability. Both CMS1 and CMS4 cell lines demonstrate decreased survival fractions at elevated temperatures. RKO cells were the most sensitive, with only 19% of the cells surviving after treatment at 43 °C. Both assays show that all five human CRC cell lines are sensitive to hyperthermia alone, although to a different extent.

### 3.2. In Colorectal Cancer Cells, the Effect of Oxaliplatin Is Temperature-Dependent; MMC is Effective at Any Temperature

**Figure 2:**
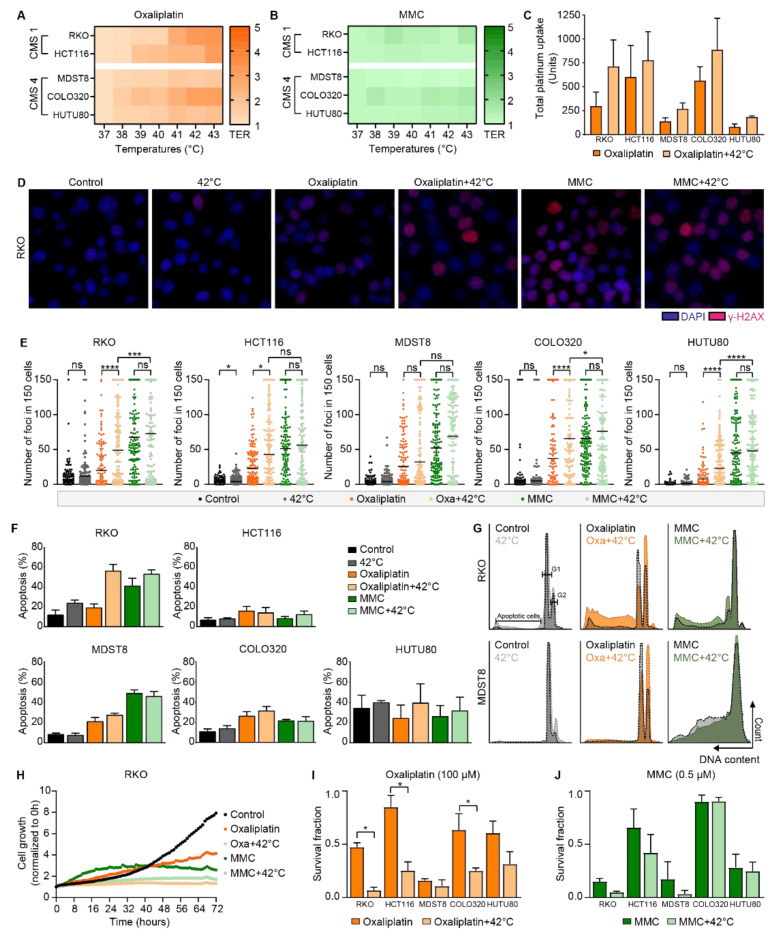
The effect of oxaliplatin is temperature-dependent, mitomycin-C (MMC) is effective at any temperature. To study the temperature-dependent effect of oxaliplatin and MMC, RKO, HCT116, MDST8, COLO320, and HUTU80 cells were exposed to oxaliplatin or MMC under hyperthermic condition for 60 min. Cell viability assays were performed at 48 h after treatment with oxaliplatin (**A**) or MMC (**B**). Thermal Enhancement Ratio (TER) values are demonstrated by a color gradient: a darker color indicates higher TER values. Data represent the mean of 3 independent experiments. (**C**) The cellular uptake of oxaliplatin was assessed by measuring platinum uptake in cells using the CyTOF at 1 h after treatment. Means ± standard error of the mean of 3 independent experiments are presented. (**D**) DNA double strand breaks were measured by nuclear γ-H2AX staining. Representative pictures of RKO cells stained for γ-H2AX are presented. (**E**) Quantification of induced foci in RKO, HCT116, MDST8, COLO320, and HUTU80 cells. Data present the number of foci in 150 cells from 3 independent experiments. Apoptosis levels (**F**) and cell-cycle distribution (**G**) were studied using the Nicoletti assay, which was performed 48 h after treatment. Means ± standard error of the mean of 3 independent experiments are presented. Representative flow charts of RKO and MDST8 cells are depicted. (**H**) Live cell imaging was used to study the growth of RKO cells after treatment. Clonogenic survival assays were performed to assess long-term cell survival 10 days after oxaliplatin (**I**) or MMC (**J**) treatment. Means ± standard error of the mean of 4 independent experiments are presented. * *p* < 0.05, *** *p* < 0.001, **** *p* < 0.0001.

To evaluate the temperature-dependent effect of the most commonly used drugs during HIPEC, cells were exposed to a range of various concentrations of oxaliplatin or MMC at clinically relevant hyperthermic temperatures (38–43 °C). First, cell viability assays were performed at 48 h after treatment. In Figure S3, the cell viability is demonstrated of RKO, HCT116, MDST8, COLO320, and HUTU80 cells after chemotherapeutic drug titrations under hyperthermic conditions. The temperature-dependent IC50 values were determined and Thermal Enhancement Ratio’s (TER) were calculated by dividing the IC50 dose at 37 °C by the IC50 dose at 43 °C. The heat map in Figure 2A demonstrates TER values at 48 h after treatment. Increased TER values of oxaliplatin are observed at elevated temperatures (41–43 °C) on both CMS1 and CMS4 cell lines, where the TER values of RKO and HCT116 cells are the highest, 3.3 and 3.1, respectively, at 43 °C (Figure S4). MMC combined with hyperthermia did not show increased TER values (Figure 2B). To investigate the effect of the treatment on healthy tissue, human skin fibroblasts were exposed to oxaliplatin and MMC alone and in combination with hyperthermia. Human bladder cancer cells were used as a positive control to confirm that MMC is synergistic with heat. Hyperthermia treatment at 42 °C decreased the cell viability with a maximum of 10% (Figure S5A). Hyperthermia in combination with oxaliplatin or MMC resulted in decreased IC50 values (Figure S5B). In Figure S5C, the TER values for MMC at 42 °C were increased in all cell lines, with 1.8 as the highest TER value in RT112. These results confirm that the synergism of MMC and heat is cell type-dependent.

Subsequently to 2D cell cultures, one normal colon and two CRC organoid models were used to validate the temperature-dependent effect of oxaliplatin and MMC in a 3D cell culture model. The cell viability of all organoids was not decreased after hyperthermia treatment at 42 °C, but hyperthermia combined with oxaliplatin treatment resulted in a more decreased cell viability compared with oxaliplatin treatment alone (Figure S5D,E). This strong temperature-dependent effect was not observed for MMC treatment. TER values for oxaliplatin are 4.6, 2.1, and 4.2, and TER values for MMC are 0.9, 0.9, and 2.3 for colon-WT, CO147, and RC511, respectively. The results in organoid cultures from normal colon tissue are comparable with the results obtained from the CRC models. The CRC organoids, which are assumed to represent the in vivo situation better than 2D cell cultures, show similar results as obtained in 2D CRC cell cultures, and therefore, the results obtained from 2D cell cultures can be considered representative.

To assess whether the cellular drug uptake is temperature-dependent, platinum uptake in the cells was measured at 1 h after oxaliplatin treatment. In Figure 2C all cell lines treated in combination with hyperthermia demonstrate an increased platinum uptake, with the highest uptake in HCT116 (750 units) and the lowest uptake in HUTU80 (78 units) and MDST8 (137 units). In Figure S6, the fold induction is demonstrated, which was significantly increased in RKO and MDST8 cells. Unfortunately, a proper method to measure the uptake of MMC was not available.

DNA DSBs were quantified by γ-H2AX staining at 24 h after treatment with oxaliplatin or MMC, and their combination with hyperthermia. Representative pictures of RKO cells after each treatment are presented in Figure 2D. DSBs were observed in all treatment groups, but significantly more for: oxaliplatin + 42 °C, MMC, and MMC + 42 °C, compared with control or hyperthermia alone (Figure 2E). Oxaliplatin + 42 °C compared with oxaliplatin alone resulted in significantly more foci in 4 out of 5 cell lines. MMC treatment alone resulted in a high number of foci, and the amount of foci was slightly increased when combined with hyperthermia. These results show that hyperthermia sensitizes oxaliplatin, resulting in more DNA DSBs. MMC is effective at both temperatures and resulted in significantly more γ-H2AX foci compared with oxaliplatin combined with hyperthermia.

The Nicoletti assay was performed to determine apoptosis and cell cycle redistribution. Treatment at 42 °C resulted in a minimal effect on the apoptotic levels of all cell lines compared with control. Oxaliplatin alone resulted in 20% apoptosis, and combination with hyperthermia resulted in an increased apoptosis of 60% in RKO cells (Figure 2F). MMC alone is as effective as MMC in combination with hyperthermia in our in vitro models, with around 50% apoptosis in RKO and MDST8 cells. The cell cycle distribution is shown in Figure 2G. The amount of cells in the G2-phase is increased after treatment with oxaliplatin and is further increased when combined with hyperthermia at 42 °C. In Figure S7, the cell-cycle distribution of all cell lines after treatment at 41–43 °C is shown. The shift towards a G2-phase starts at 41 °C and increases at higher temperatures. After treatment with MMC, there is a G2-arrest observed after treatment at any temperature.

We also verified whether our results might be influenced by the autonomous cell cycle and doubling time of the CRC cells studied. Figure S2 represents the characteristics of the CRC cell lines used, including doubling time. Doubling time of the cell lines varied between 21 and 32 h. RKO cells are the most sensitive to hyperthermia with a doubling time of 25 h, whereas MDST8 cells, a cell line less sensitive to hyperthermia, have a comparable doubling time of 27 h. Thus, doubling time was not correlated with sensitivity hyperthermia.

BrdU was incorporated in cells at 16–24 h after treatment to confirm the results obtained by the Nicoletti assay. Unfortunately, cells treated with oxaliplatin or other chemotherapeutic agents did not allow the incorporation of BrdU (Figure S8). These results suggest that cells do not proliferate within this time frame. Live imaging over time was used to measure the confluency until 3 days after treatment to study cell growth. In Figure 2H, the cell growth of RKO cells after treatment with oxaliplatin or MMC with and without hyperthermia is studied. Oxaliplatin or MMC treatment alone decreased cell growth, compared with control. When combined with hyperthermia, RKO cells did not grow within 72 h.

To investigate long-term survival after in vitro HIPEC treatments, clonogenic survival assays were performed at 10 days after treatment. Survival fractions after treatment with oxaliplatin combined with hyperthermia decreased survival, with significant lower survival in HCT116 and COLO320 cells (Figure 2I). The survival after MMC treatment is presented in Figure 2J and demonstrates that MMC without hyperthermia results in decreased survival. In Figure S9, the results of clonogenic survival assays performed with all five drugs and cell lines at 41, 42, or 43 °C are shown. IC50 values and TER values obtained by clonogenic survival assays are presented in Figure S10.

In summary, all these results show a temperature-dependent efficacy for oxaliplatin and show that MMC is effective at any temperature.

### 3.3. The Effect of Cisplatin and Carboplatin Is Also Temperature-Dependent in Colorectal Cancer Cells

**Figure 3:**
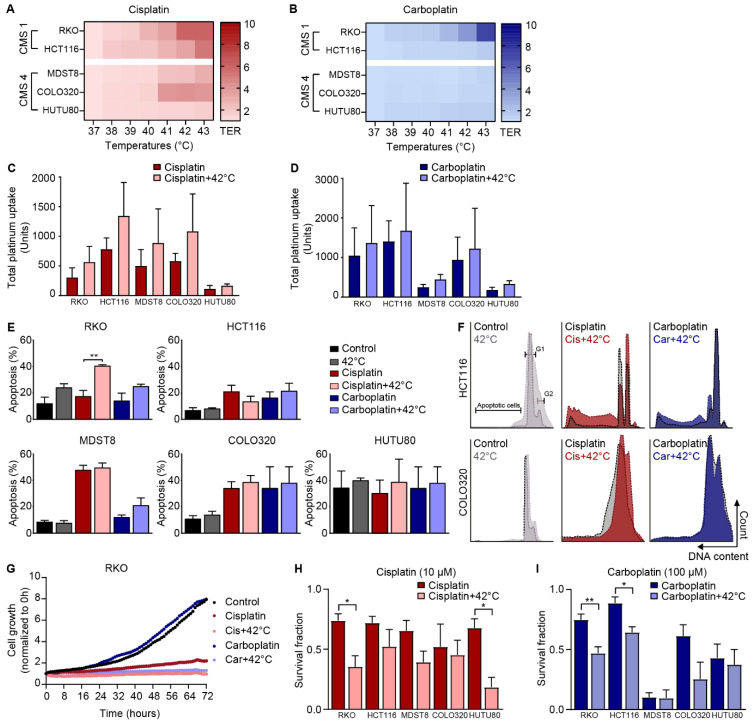
The effect of cisplatin and carboplatin is also temperature-dependent in colorectal cancer cells. To study the temperature-dependent effect of other platinum-based drugs, RKO, HCT116, MDST8, COLO320, and HUTU80 cells were exposed to cisplatin or carboplatin, under hyperthermic condition for 60 min. Cell viability assays were performed 48 h after treatment with cisplatin (**A**) or carboplatin (**B**). Thermal Enhancement Ratio (TER) values are demonstrated by a color gradient: a darker color indicates higher TER values. Data represent the mean of 3 independent experiments. The cellular uptake of cisplatin (**C**) and carboplatin (**D**) was assessed by measuring platinum uptake in cells using the CyTOF at 1 h after treatment. Means ± standard error of the mean of 3 independent experiments are presented. Apoptosis levels (**E**) and cell cycle distribution (**F**) was studied using the Nicoletti assay, which was performed 48 h after treatment. Representative flow charts of HCT116 and COLO320 cells are presented. (**G**) Live cell imaging was used to study the growth of RKO cells after treatment. Clonogenic survival assays were performed to assess long-term cell survival at 10 days after cisplatin (**H**) or carboplatin (**I**) treatment. Means ± standard error of the mean of 4 independent experiments are presented. * *p* < 0.05, ** *p* < 0.01.

The temperature-dependent effect of two other platinum-based drugs, cisplatin and carboplatin, were evaluated. In the heat map in Figure 3A, the TER values of cisplatin are shown. The TER values were increased at higher temperatures starting at 41 °C to 3.5, 2.8, and 3.9 in RKO, HCT116, and COLO320 cells, respectively (Figure S4). Carboplatin showed only increased TER values in RKO cells (Figure 3B) with ratios of 2.5, 3.8, and 7.2 at 41, 42, and 43 °C, respectively.

To study whether temperature influences the cellular drug uptake, platinum uptake was measured after treatment with cisplatin or carboplatin. In all cell lines, treatment including hyperthermia resulted in more platinum uptake after cisplatin and carboplatin treatment, with the highest uptake in HCT116 cells and the lowest in MDST8 and HUTU80 cells (Figure 3B,C). The addition of hyperthermia to the drugs resulted in almost 2-fold more platinum uptake in all cell lines (Figure S6B,C).

The Nicoletti assay was performed to determine apoptosis and cell cycle redistribution. Treatment at 42 °C revealed a minimal effect on the apoptosis levels of all cell lines compared with control. Cisplatin alone resulted in 20% apoptosis and combination with hyperthermia resulted in an increased apoptosis of 40% in RKO cells (Figure 3E). In the other cell lines, the same trend was observed. Carboplatin combined with hyperthermia resulted in increased apoptosis, although these differences were not significant. The cell cycle distribution of HCT116 and COLO320 cells are presented in Figure 3F and show more cells in G2-phase after treatment with cisplatin or carboplatin, which was even more increased when combined with hyperthermia.

Cell growth was assessed using live imaging and the results are shown in Figure 3G. RKO cells did not proliferate within 72 h after treatment with cisplatin, and when combined with hyperthermia. Carboplatin treatment did not influence the growth of cells, but growth was decreased if combined with hyperthermia.

In Figure 3H, the results of clonogenic survival assays after cisplatin treatment are shown. Hyperthermia combined with cisplatin resulted in decreased survival compared with cisplatin treatment alone. Survival fractions were decreased after carboplatin treatment, and a significant decrease was observed in both CMS1 cell lines, when combined with hyperthermia (Figure 3I).

These experiments show that cisplatin and, to a lower extent, carboplatin have a temperature-dependent effect comparable with oxaliplatin.

### 3.4. 5-FU, as a Systemic Drug in Combination With HIPEC Treatment

**Figure 4:**
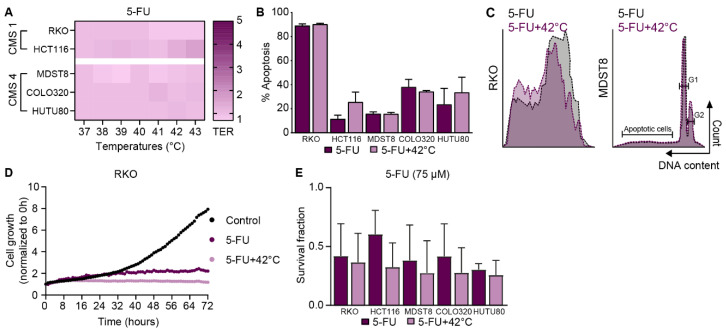
5-Fluorouracil (5-FU), as a systemic drug in combination with hyperthermic intraperitoneal chemotherapy (HIPEC) treatment. (**A**) Thermal Enhancement Ratio (TER) values of 5-FU obtained from cell viability assay are demonstrated by a color gradient: a darker color indicates higher TER values. The mean of 3 independent experiments are presented. Apoptosis levels (**B**) and cell cycle distribution (**C**) was studied using the Nicoletti assay, which was performed at 48 h after treatment with 5-FU. (**D**) Live cell imaging was used to give more insights about the growth of the cells after treatment. (**E**) Clonogenic survival assays were performed to assess long-term cell survival at 10 days after 5-FU treatment. Means ± standard error of the mean of 4 independent experiments are presented.

The additional effect of hyperthermia to 5-FU for the application of HIPEC was tested in CRC cell lines. The TER values of 5-FU were not increased at elevated temperatures (Figure 4A). Treatment of cells with 5-FU resulted in apoptosis, and combination with hyperthermia did not affect the apoptotic levels significantly (Figure 4B). RKO cells were the most sensitive, resulting in almost 90% cell death. Cell cycle distribution was not affected by 5-FU treatment alone or in combination with hyperthermia (Figure 4C). Cell growth was assessed using live imaging (Figure 4D). RKO cells treated with 5-FU proliferated 4 times less compared to control, independent of temperature. In Figure 4E, survival fractions at 10 days after treatment with 5-FU do not show significant changes when combined with hyperthermia.

These results demonstrate that 5-FU is an effective drug for human CRC cells, without a temperature-dependent effect.

### 3.5. P53 Status Does Not Affect the Sensitivity of CRC for Hyperthermia

**Figure 5:**
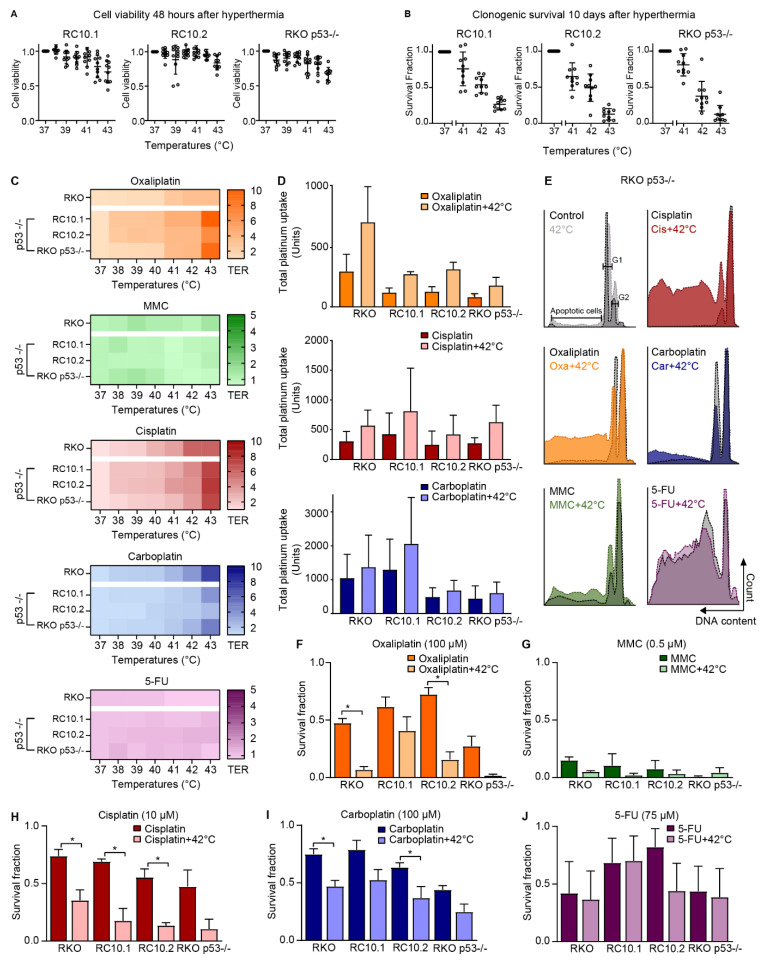
P53 status does not affect the sensitivity of colorectal cancer (CRC) to hyperthermia. P53 protein function plays a central role in the arrest of the cell cycle in G1 phase after DNA damage. To study whether p53 status influences the sensitivity for HIPEC treatment, RC10.1, RC10.2, and RKO p53−/− cells with a decrease of p53 levels and function were used. These cells were treated with hyperthermia alone to assess the cell viability (**A**) 48 h after treatment and clonogenicity (**B**) was assessed 10 days after treatment by clonogenic survival assays. For both experimental set ups, cells at 37 °C represent the untreated controls. The mean of 10 independent experiments are presented. (**C**) Thermal Enhancement Ratio (TER) values obtained from cell viability assays are demonstrated by a color gradient: a darker color indicates higher TER values. The mean of 3 independent experiments are presented. (**D**) The cellular uptake of oxaliplatin, cisplatin, and carboplatin was assessed by measuring the platinum uptake using the CyTOF. Means ± standard error of the mean of 3 independent experiments are presented. (**E**) Representative flow charts of the Nicoletti assay to assess apoptotic levels and cell cycle distribution. Clonogenic survival assays were performed to assess long-term cell survival 10 days after treatment with oxaliplatin (**F**), MMC (**G**), cisplatin (**H**), carboplatin (**I**), and 5-FU (**J**). Means ± standard error of the mean of 4 independent experiments are presented. * *p* < 0.05.

The effect of hyperthermia on p53 status was studied in RKO cells with wild-type p53 and in cells without functional p53, e.g., RKO cells transfected with HPV16-E6-1 (RC10.1) and HPV16-E6-2 (RC10.2), and in a dominant negative p53 mutant (RKO p53−/−). Cell viability (Figure 5A) and clonogenic survival assays (Figure 5B) were performed after cells were exposed to hyperthermia, chemotherapeutic drugs, or a combination of both. Hyperthermia alone decreased the cell viability and clonogenicity in all cell lines. Increased TER values for cisplatin, oxaliplatin, and carboplatin at temperatures of 41 °C and higher are seen in heat maps (Figure 5C). MMC and 5-FU did not demonstrate a temperature-dependent effect at 48 h after treatment.

Increased platinum uptake at elevated temperatures was observed (Figure 5D), which is similar to the platinum uptake in wild-type RKO cells. The platinum uptake is presented in fold induction (Figure S6D–F). Hyperthermia resulted in approximately 2 times more platinum uptake when treated with oxaliplatin, cisplatin, or carboplatin in all cell lines.

To examine whether p53 status is relevant for cell cycle distribution, the Nicoletti assay was performed. An induction of G2-phase after treatment with oxaliplatin, MMC, cisplatin, and carboplatin was observed in RKO p53 −/− cells (Figure 5E). Combination treatment with hyperthermia resulted in elevated G2 levels and increased apoptosis compared with cells that were exposed to drugs only. Similar results were obtained for RC10.1 and RC10.2 cells and comparable with wild-type RKO cells (Figure S7).

Treatment with oxaliplatin, cisplatin, and carboplatin resulted in decreased survival fractions, and when combined with hyperthermia at 42 °C, a decrease was observed in both CMS1 and CMS4 cell lines (Figure 5F,H,I). Addition of hyperthermia did not result in decreased survival compared with MMC or 5-FU exposure alone (Figure 5G,J).

In summary, these results show that the p53 status does not affect the cell viability, platinum uptake, cell cycle distribution, or clonogenicity, after in vitro HIPEC treatment, compared with RKO cells with wild-type p53.

## 4. Discussion

In the present study, HIPEC was mimicked in an in vitro setting by exposing eight different human CRC cell lines to cisplatin, carboplatin, oxaliplatin, MMC, or 5-FU at clinically relevant hyperthermic temperatures: 38–43 °C for 60 min. Our data demonstrate that platinum-based drugs are effective for CRC at temperatures above 41 °C, suggesting that they are, in principle, suitable for use during HIPEC, which is always performed at more elevated temperatures. We found that hyperthermia increases platinum-based drug uptake, results in G2-arrest, increases DNA DSBs, increases apoptotic levels, and decreases cell growth. These results confirm that platinum-based drugs are synergistic with heat with a clear dose-effect relationship. Interestingly, a relation with level of platinum uptake could not be established, and a higher platinum uptake did not result in more apoptosis. Carboplatin was the least synergistic with heat of the three platinum-based drugs.

Our data demonstrate that each cell line responds differently to hyperthermia. RKO cells are the most sensitive, whereas HCT116 and CMS4 cell lines are sensitive, but to a lesser degree. Previous research by Luo et al. concluded that HCT116 cells are sensitive to hyperthermia [31]. Apoptosis levels of 15% and 25% were observed after a 2- and 4-h exposure to 42 °C, respectively. Major differences between our experimental design and this previous study are the exposure time to hyperthermia and the incubation time prior to analysis. In our study, a 60-min exposure to hyperthermia is used and 24, 48, and 10 days after treatment cell survival is assessed with several assays, compared with only 2 or 4 h prior to analysis. This is likely to explain the difference in results reported in the study by Luo et al. and our study, since it is know that cell death is correlated with exposure time to hyperthermia [32].

MMC, one of the main drugs used for HIPEC procedures in CRC, resulted in increased apoptosis, G2-arrest, and decreased cell growth at any temperature. Our in vitro results show that MMC is not synergistic with heat in CRC cells, which is in line with an in vivo study in mice containing spontaneous murine fibrosarcoma which demonstrated that cisplatin did, and 5-FU and MMC did not result in a tumor growth delay after HIPEC treatment at 41.5 °C compared with chemotherapy alone [33]. On the other hand, Sorensen et al. demonstrated on rats bearing pseudomyxoma peritonei that 90 min heating and MMC at 41 °C enhanced survival, when compared with treatment with MMC alone [34]. In line with our results obtained in bladder cancer cells, Van der Heijden and colleagues found a clear synergistic effect of MMC and hyperthermia (43 °C) in four human bladder cancer cell lines [35]. The presence or absence of synergism may thus be dependent on the cell line origin, the temperatures, and the duration of heating.

The platinum uptake in cells was determined using CyTOF. Determination of the MMC uptake for comparison would provide relevant insights, but unfortunately a reliable method for detection and quantification of MMC is lacking. Various methods have been described in the literature, such as high performance liquid chromatography (HPLC), either with or without UV detection [36,37]. Another option could be using solid phase extraction (SPE) as sample preparation to determine MMC in human plasma and urine [38]. However, all these methods lack a thorough validation, and therefore, we did not include measurements of the MMC uptake in this study.

Besides 2D cell cultures, we used human CRC organoid models to validate our findings. As presented in the results, hyperthermia treatment alone does not affect the growth of CRC or normal colon organoids. Treatment with oxaliplatin or MMC resulted in decreased cell viability, with oxaliplatin showing a strong temperature-dependent effect. The IC50 values for oxaliplatin found in our study, are slightly lower than the clinical concentrations in two out of five cell lines, and higher in the organoid models. IC50 values of MMC are much lower than clinical concentrations in four out of five cell lines, in particular within CMS4 cell lines and the CRC organoid model. Our results suggest, similar to the results of Ubink and colleagues [39], that MMC is more effective in reducing cell viability than oxaliplatin at clinically relevant concentrations, when treated for exposure times matching current HIPEC regimes. Ubink et al. treated peritoneal metastases-derived organoids with oxaliplatin (30 min) or MMC (90 min), both at 42 °C [39]. Results showed that IC50 values were higher than the median clinical dose in two of five organoid lines for MMC, and all five lines for oxaliplatin, suggesting that MMC is more effective when using a 90-min exposure time, which is three times longer than the exposure time used for oxaliplatin. In our in vitro data, the highest IC50 values were found in HCT116 cells, the only cell line with a KRAS pathway mutation, which is associated with decreased survival in PMCRC patients treated with oxaliplatin [40,41].

Surprisingly, outcome of recent randomized multi-center HIPEC trials using platinum-based drugs was negative. A 30-min oxaliplatin-based HIPEC treatment did not improve overall survival (OS) or peritoneal metastasis-free survival of patients with PMCRC. The Prodige-7 trial, a French multicenter trial randomizing between CRS alone or CSR with HIPEC, included patients with histologically proven PMCRC who had initially been treated with systemic chemotherapy, mostly oxaliplatin-based [42]. Median overall survival was similar: 41.2 months in the CRS alone-arm vs. 41.7 months in the CRS/HIPEC-arm. Post-operative mortality was not different, but morbidity at 60 days after treatment was higher in the CRS/HIPEC-arm (24.1% vs. 13.6%; *p* = 0.03).

In the Dutch randomized multicenter COLOPEC trial in patients with stage II-III colon cancer with a c/pT4 or perforation, peritoneal metastasis-free survival was assessed after receiving 30 min oxaliplatin-based HIPEC following adjuvant chemotherapy [43]. After 18 months, peritoneal metastasis-free survival was not significantly improved (76.2% in control-arm vs. 80.9% in HIPEC-arm), which can partly be attributed to bias favoring the control arm, as metastases could be detected earlier during the HIPEC in the experimental arm. These negative outcomes resulted in a discussion about the use of HIPEC for the treatment of PMCRC [44,45]. Both trials used the ‘French’ HIPEC protocol; a high dose of oxaliplatin (460 mg/m^2^) combined with hyperthermia (42–43 °C), with a duration of 30 min. The concentration of the drug is much higher than IC50 doses demonstrated in several in vitro studies, though with an exposure of just 30 min [46,47,48]. Oxaliplatin is a blistering agent, a high dose can lead to chemical irritations of the peritoneum leading to encapsulating sclerosing peritonitis [45]. The significantly higher morbidity in the HIPEC arm in the Prodige-7 trial can be a result of the high concentration of oxaliplatin. The reported negative treatment outcomes are surprising, as we found in our in vitro data a significant to strong effect on clinically representative CMS1 and CMS4 CRC cell lines after oxaliplatin exposure, with a duration of 60 min, suggesting that a longer exposure (>30 min) of oxaliplatin could lead to better clinical outcome.

Since the negative outcome of oxaliplatin-based HIPEC for PMCRC patients, MMC-based HIPEC is considered as a standard regime for the treatment of PMCRC. Unfortunately, studies comparing oxaliplatin or MMC-based HIPEC did not show a clear benefit for either regime in overall survival or disease free survival in patients with PMCRC [49,50]. Although no meaningful comparison could be made because of the essential differences between the procedures (e.g., duration, temperature), no preference towards one of the methods could be demonstrated [50]. Oxaliplatin was reported to result in equally high morbidity rates as MMC, but MMC often results in neutropenia [49].

The relatively short (30–120 min) duration of HIPEC motivates the use of cell cycle non-specific drugs. 5-FU, a cell cycle-specific drug, requires a long exposure time to induce cell death and is therefore used as a systemic treatment before or after the HIPEC procedure [51,52]. However, our data show that short (60 min) exposure to 5-FU does have a tumor-suppressive effect on CRC cells. We observed increased apoptotic levels and decreased cell growth, but the effects were not temperature-dependent. Moreover, the cell cycle distribution was unchanged after a 60-min treatment with 5-FU. Murata et al. demonstrated that 5-FU was concentration-dependent in gastric cancer cells, and combined with cisplatin or MMC, the tumor cell growth effect was significantly inhibited compared with monotherapy [53]. Additionally, in CRC cells, the administration of MMC and 5-FU resulted in suppressed tumor cell proliferation compared with either agent used alone [54]. 5-FU alone or in combination with MMC can be a promising drug for the application of HIPEC for PMCRC patients.

Our data show a correlation between a higher oxaliplatin uptake and a higher number of DNA DSBs. The highest oxaliplatin uptake was observed in RKO, HCT116, and COLO320 cells, when combined with hyperthermia. In these cell lines, the highest number of γ-H2AX foci was observed 24 h after treatment. The apoptotic levels, 48 h after treatment, did not show a clear correlation with the previous observations. Although most of the DNA repair is within 24 h after treatment, our data suggest that the dominant DNA repair mechanism differs per cell line. Both oxaliplatin and MMC induce DNA DSBs. Those breaks can be repaired via two major pathways: homologous recombination (HR) and non-homologous end joining (NHEJ). One of the effects of hyperthermia is degradation of BRCA2, an important protein involved in HR DNA repair. Subsequently, HR is temporarily inhibited, which results in decreased cell survival. However, the decrease of BRCA2 protein levels after hyperthermia require temperatures above 41 °C, with showing an increased long-term BRCA2 depletion at higher temperatures and longer exposure time [55,56]. Remarkably, our data show that inhibiting DNA repair pathways by hyperthermia does not increase the amount of MMC-induced DNA DSBs. Earlier studies demonstrated a remarkable and temperature-dose dependent increase in sensitivity of FSa-II cells for cisplatin and oxaliplatin above 41 °C [57]. TER also increases in a temperature-dependent manner for most cell lines included in our study, particularly at temperatures above 41 °C, though the magnitude of this increase differs significantly for different cell lines and different platinum-based agents. This finding is in agreement with the dose-effect-relationships reported earlier [55,56].

Transcription factor p53 plays an important role in controlling apoptosis, DNA repair, and cell cycle arrest. P53 is inactivated at elevated temperatures (>41 °C), which makes the p53 pathway heat sensitive [27,58]. In vitro experiments showed that inactivation of p53 protects human colorectal carcinoma cells against hyperthermia-induced cytotoxicity and apoptosis [58]. However, our data show no differences in cell viability, clonogenicity, cell cycle distribution, or platinum uptake after in vitro HIPEC treatment in RKO cells with or without inactivation of p53. Van Bree and colleagues also demonstrated that hyperthermia-enhanced cytotoxicity of cisplatin does not depend on p53 function [59]. These findings combined with our data suggest that p53 status is not important for the hyperthermia-induced cytotoxicity and apoptosis of platinum-based drugs, MMC or 5-FU, thus further research on the p53 status on more CRC cell lines and patient biopsies is needed to confirm the irrelevance of p53 status for HIPEC for PMCRC patients.

The molecular mechanism behind the synergism between hyperthermia and chemotherapeutic drugs has not yet been unraveled. Our in vitro data suggest that p53 is not playing an essential role in this mechanism. Moreover, although our results demonstrate a correlation between the platinum uptake and the amount of DNA DSBs, interestingly, a correlation with more apoptosis could not be established. Most DNA damage is repaired within 24 h after treatment, but the timeframe of 24–48 h after treatment should be studied to determine whether the dominant DNA repair mechanism differs per cell line. Another important issue that should be addressed in further research is how the uptake of the drugs is regulated and by which cellular pathway. For the uptake of platinum-based drugs, the copper transporter-1 (CTR-1) and the organic cation transporter 1–3 (OCT1–3) have been described to be important [60,61]. Cui et al. demonstrated that different human CRC cell lines express high levels of CTR-1 protein in similar localization patterns, despite the different sensitivity to oxaliplatin [60]. Unfortunately, the transporters important for the uptake of MMC are still not fully known. Unravelling the molecular mechanism of these transporters and to assess the effect of hyperthermia on the expression of these transporters should provide new insights and can lead to a more effective HIPEC treatment regime.

## 5. Conclusions

**Figure 6:**
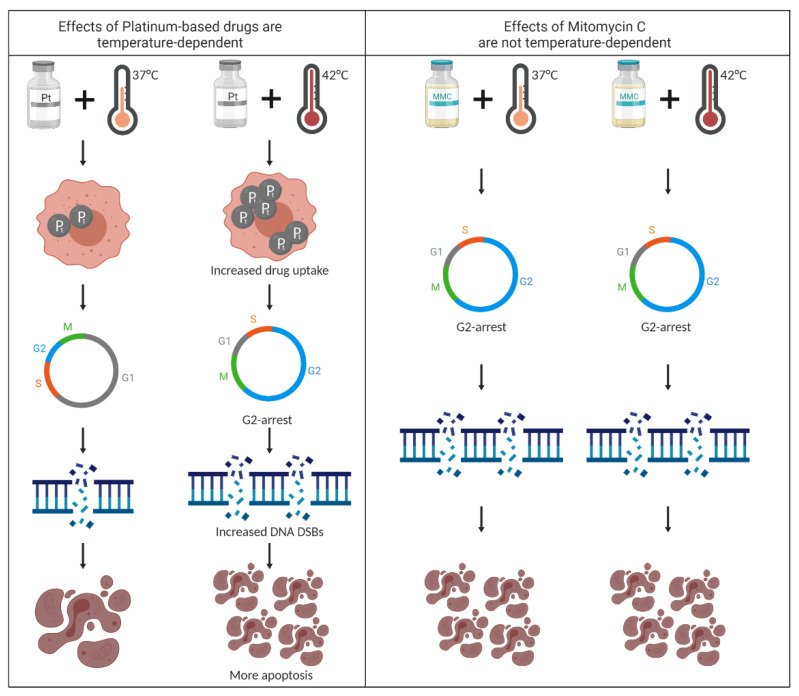
Graphical conclusion. (**A**) Platinum-based drugs require hyperthermia to induce significantly more DNA double strand breaks (DSBs), G2 cell cycle arrest, and increased apoptosis in colorectal cancer cells. (**B**) MMC treatment, even without hyperthermia, induces DNA DSBs, results in G2-arrest and leads to high apoptotic levels in colorectal cancer cells. Created with Biorender.com.

In conclusion, in our in vitro study, we demonstrate that platinum-based drugs and MMC are effective drugs for CRC cells and could be suitable for HIPEC, even when hyperthermia does not sensitize MMC in an in vitro setting (Figure 6). Our data indicate that a short exposure of 60 min is effective, and if drugs are synergistic with heat, temperatures above 41 °C should be applied to achieve a sufficient tumor-suppressive effect. The obtained biological data on the temperature-dependent effectiveness of the chemotherapeutic agents in CMS1 and CMS4 cells can form a basis for the development of CMS-type based or patient specific protocols for the application of HIPEC. Quantifying the effect for each HIPEC parameter is necessary. Our in vitro study provides insights on the effects of drug exposure leading to cell death in a model in which all cancer cells are equally exposed to the drug at a homogenous treatment temperature. This provides relevant basic insight on the temperature-dependent effect for specific drugs, showing that a temperature difference of 1 °C has a great impact on the effectivity. However, during the application of HIPEC in patients, the temperature and drug distribution is disturbed by the organs in the peritoneum, resulting in temperature and drug concentration inhomogeneities. Therefore, additional in vivo studies, with realistic HIPEC conditions including temperature and drug inhomogeneities, are necessary to define the optimal treatment regime for HIPEC. Animal models should be used to establish the drug-, temperature-, and duration-dependent tissue drug penetration during HIPEC, adding that these animal data should constitute a stronger scientific foundation for a biological rationale for establishing standardized HIPEC protocols for PMCRC patients.

## Supplementary Materials

The following are available online at https://www.mdpi.com/2073-4409/9/8/1775/s1, Figure S1: Overview of the in vitro HIPEC treatment set-up and performed experiments. Created with Biorender.com; Figure S2: Table with information about CMS classification, doubling time, MSI status and pathway mutations of the used eight human colorectal cancer cell lines (A), human bladder cancer cell lines (B), human colorectal cancer organoid models (C) and normal cell lines (D); Figure S3: Cell viability assays were performed 48 h after hyperthermia treatment at 38, 39, 40, 41, 42 and 43 °C. Cells at 37 °C represent the control temperature. The mean of 3 independent experiments are presented; Figure S4: IC50 and TER values obtained from cell viability performed 48 h after hyperthermia treatment at 38, 39, 40, 41, 42 and 43 °C. Cells at 37 °C represent the control temperature. The mean of 3 independent experiments are presented; Figure S5: AG1522, T24 and RT112 cells were treated with hyperthermia alone (A) and in combination with oxaliplatin or MMC (B) to assess the cell viability 48 h after treatment. IC50 and TER values (C) obtained from cell viability performed 48 h after hyperthermia treatment at 42 °C. Colon-WT, CO147 and RC511 organoids were treated with hyperthermia alone (D) and in combination with oxaliplatin or MMC (E) to assess the cell viability 48 h after treatment. IC50 and TER values (F) obtained from cell viability performed 48 h after hyperthermia treatment at 42 °C. Cells at 37 °C represent the control temperature. The mean of 3 independent experiments are presented; Figure S6: The cellular uptake of oxaliplatin, cisplatin and carboplatin was assessed by measuring the platinum uptake using the CyTOF. Data was normalized to chemotherapeutic drug exposure alone to obtain fold induction. Means ± standard error of the mean of 3 independent experiments are presented. * *p* ≤ 0.05, ** *p* ≤ 0.01, *** *p* ≤ 0.001; Figure S7: Representative flow charts of the Nicoletti assays performed at 48 h after treatment of RKO, HCT116, MDST8, COLO320, HUTU80, RC10.1, RC10.1 and RKO p53−/− cells; Figure S8: Representative BrdU staining on RKO cells performed at 16–24 h after treatment; Figure S9: Long-term cell survival was assessed by using clonogenic survival assays performed 10 days after treatment at 41, 42, 43 °C. Cells at 37 °C represent the control temperature. Means ± standard error of the mean of 4 independent experiments are presented; Figure S10: IC50 and TER values obtained from clonogenic survival assays performed 10 days after treatment at 41, 42, 43 °C. Cells at 37 °C represent the control temperature. Means ± standard error of the mean of 4 independent experiments are presented. Figure S11: Statistical *p*-values main figures.
