# Supplementary material for: The Temperature-Dependent Effectiveness of Platinum-Based Drugs Mitomycin-C and 5-FU during Hyperthermic Intraperitoneal Chemotherapy (HIPEC) in Colorectal Cancer Cell Lines
Source: Cells. 2020 Jul 25;9(8):1775. doi: 10.3390/cells9081775 (PMC7464333; doi:10.3390/cells9081775)
Supplement: Supplementary file 1 [file cells-09-01775-s001.pdf]

Supplementary Figure 1

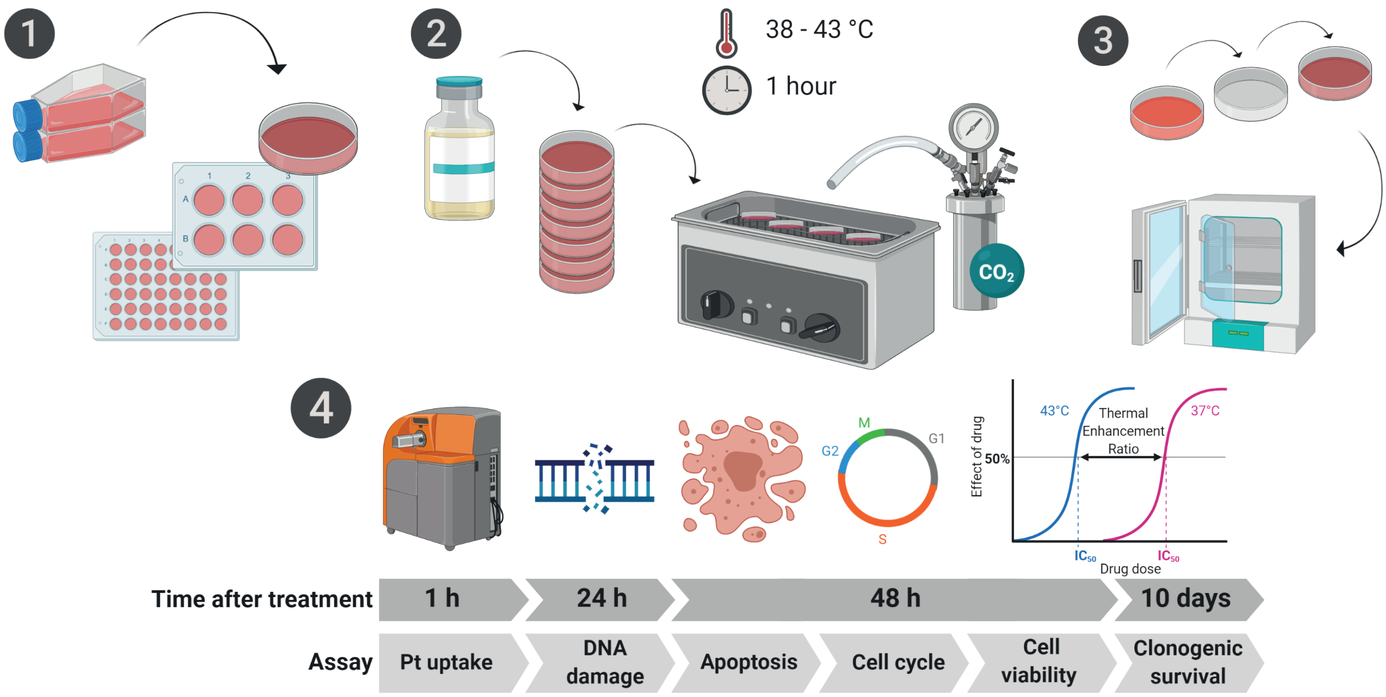

Supplementary Figure 2

A

Subtype	p53 status	Cell line	DT	MSI	WNT	p53	KRAS	RTK/RAS	BRAF	PI3K	TGF-β	BRCA2	Characteristics
CMS1	p53-WT	RKO	25 h	MSI	No	No	No	Yes	Yes	Yes	No	Yes	
CMS1	p53-WT	HCT116	20 h	MSI	Yes	No	Yes	Yes	No	Yes	No	Yes	
CMS1	p53 mutant	RC10.1	27 h	MSI	No	Yes	No	Yes	Yes	Yes	No	Yes	HPV16-E6-1
CMS1	p53 mutant	RC10.2	28 h	MSI	No	Yes	No	Yes	Yes	Yes	No	Yes	HPV16-E6-2
CMS1	p53 mutant	RKO p53-/-	27 h	MSI	No	Yes	No	Yes	Yes	Yes	No	Yes	Dominant negative p53 mutant
CMS4	p53-WT	MDST8	27 h	MSS	Yes	No	No	Yes	Yes	No	No	No	
CMS4	p53-WT	COLO320	32 h	MSS	No	Yes	No	Yes	No	No	Yes	No	
CMS4	p53-WT	HUTU80	21 h	MSS	Yes	No	No	No	No	No	No	No	Derived from small intestine

CMS: Concensus Molecular Subtype; WT: Wild-Type; DT: Doubling Time; MSI: Microsatellite Instable; MSS: Microsatellite Stable

Pathway mutation
Yes (green)
No (red)

B

	Cell line	DT	Molecular Subtype	FGFR3	PIK3CA	KDM6A	p53	TERT	PIK3CA	KRAS	Characteristics
Bladder	T24	20 h	Basal	Yes	No	Yes	Yes	Yes	Yes	No	
Bladder	RT112	25 h	Luminal	No	No	No	Yes	Yes	Yes	No	

WT: Wild-Type; DT: Doubling Time;

Pathway mutation
Yes (green)
No (red)

C

	Cell line	DT	MSI	p53	KRAS	PI3KCA	BRAF	NRAS
CMS4	RC511	N/A	MSS	No	No	Yes	No	No
CMS4	CO147	N/A	MSS	Yes	No	No	Yes	No

Pathway mutation
Yes (green)
No (red)

CMS: Concensus Molecular Subtype; WT: Wild-Type; DT: Doubling Time; MSI: Microsatellite Instable; N/A: not applicable; MSS: Microsatellite Stable

D

	Cell line	DT	MSI	Origin
Healty	AG1522	20 h	N/A	Fibroblast from the human skin
Healty	Colon-WT	N/A	N/A	Healthy part the human colon

WT: Wild-Type; DT: Doubling Time; N/A: not applicable; MSI: Microsatellite Instable;

Supplementary Figure 3

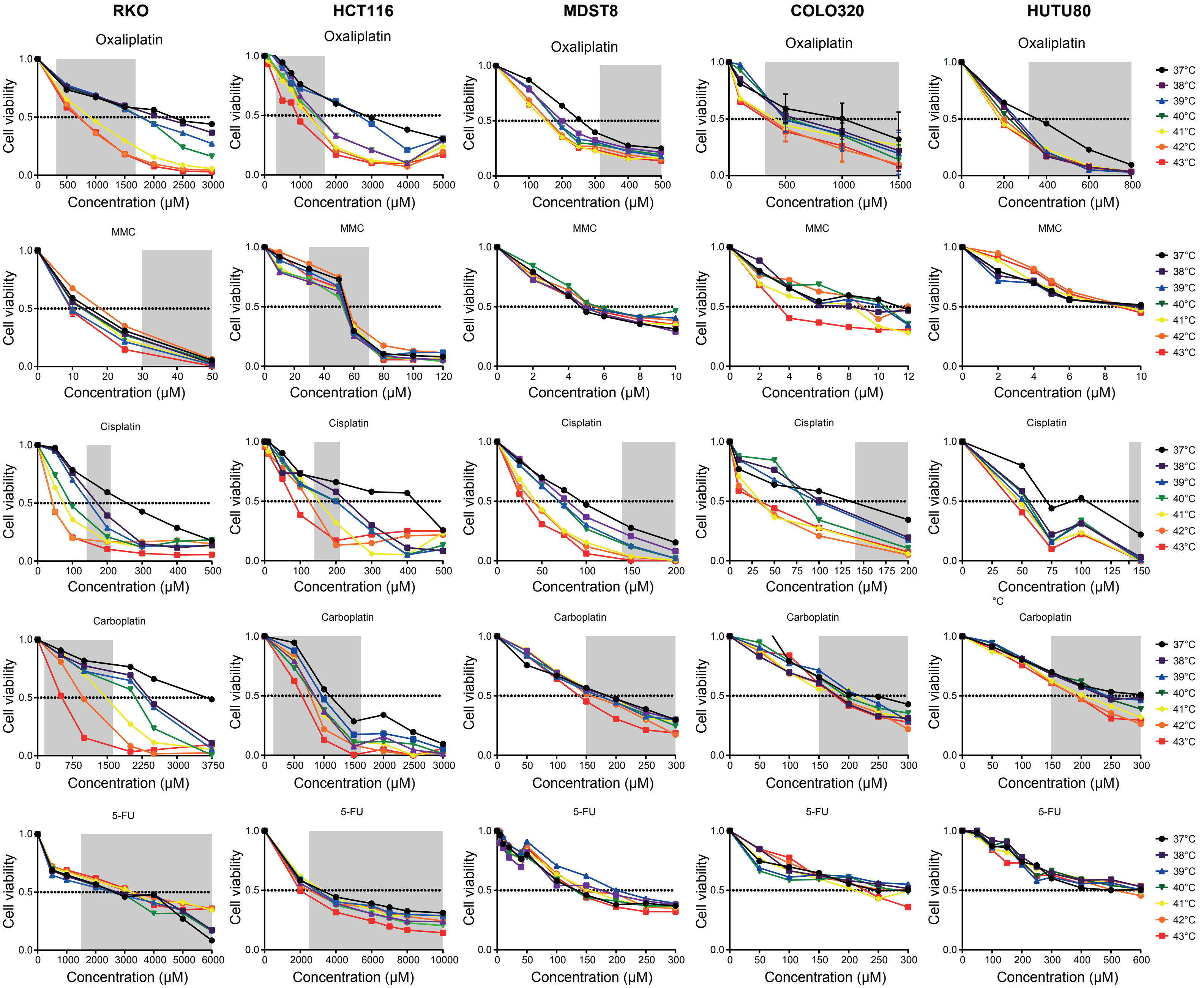

Supplementary Figure 4

Oxaliplatin	Temperature (°C)	37	38	39	40	41	42	43
RKO	IC50 (µM)	2320	2115	1770	1770	910	725	695
	TER	1.0	1.1	1.3	1.3	2.6	3.2	3.3
HCT116	IC50 (µM)	2850	2639	1486	1428	1252	1214	920
	TER	1.0	1.1	1.9	2.0	2.3	2.3	3.1
MDST8	IC50 (µM)	256	200	183	183	150	158	150
	TER	1.0	1.3	1.4	1.4	1.7	1.6	1.7
COLO320	IC50 (µM)	1000	613	500	525	405	337	330
	TER	1.0	1.6	2.0	1.9	2.5	3.0	3.0
HUTU80	IC50 (µM)	357	248	260	226	180	194	180
	TER	1.0	1.4	1.4	1.6	2.0	1.8	2.0

MMC	Temperature (°C)	37	38	39	40	41	42	43
RKO	IC50 (µM)	14.7	13.2	9.7	12.5	12.5	17.9	9.7
	TER	1.0	1.1	1.5	1.2	1.2	0.8	1.5
HCT116	IC50 (µM)	56	55	53	53	56	56	55
	TER	1.0	1.0	1.1	1.1	1.0	1.0	1.0
MDST8	IC50 (µM)	4.7	5.0	5.0	5.6	5.0	5.7	5.0
	TER	1.0	0.9	0.9	0.8	0.9	0.8	0.9
COLO320	IC50 (µM)	11.5	8.3	10.2	10.5	8.3	8.9	8.0
	TER	1.0	1.4	1.1	1.1	1.4	1.3	1.4
HUTU80	IC50 (µM)	10	10	10	10	9.3	9.1	8.7
	TER	1.0	1.0	1.0	1.0	1.1	1.1	1.2

Cisplatin	Temperature (°C)	37	38	39	40	41	42	43
RKO	IC50 (µM)	256	172	148	93	73	43	43
	TER	1.0	1.5	1.7	2.8	3.5	6.0	6.0
HCT116	IC50 (µM)	422	228	200	200	153	124	81
	TER	1.0	1.9	2.1	2.1	2.8	3.4	5.2
MDST8	IC50 (µM)	93	79	70	70	43	40	31
	TER	1.0	1.2	1.3	1.3	2.2	2.3	3.0
COLO320	IC50 (µM)	134	103	98	84	34	33	34
	TER	1.0	1.3	1.47	1.6	3.9	4.1	3.9
HUTU80	IC50 (µM)	71	56	54	51	48	48	42
	TER	1.0	1.3	1.3	1.4	1.5	1.5	1.7

Carboplatin	Temperature (°C)	37	38	39	40	41	42	43
RKO	IC50 (µM)	3635	2385	2308	2096	1460	967	502
	TER	1.0	1.5	1.6	1.7	2.5	3.8	7.2
HCT116	IC50 (µM)	1095	972	845	845	845	767	633
	TER	1.0	1.1	1.3	1.3	1.3	1.4	1.7
MDST8	IC50 (µM)	186	186	170	170	186	158	137
	TER	1.0	1.0	1.1	1.1	1.0	1.2	1.4
COLO320	IC50 (µM)	230	185	216	200	216	184	177
	TER	1.0	1.2	1.1	1.2	1.1	1.3	1.3
HUTU80	IC50 (µM)	300	239	240	237	204	189	189
	TER	1.0	1.3	1.3	1.3	1.5	1.6	1.6

5-FU	Temperature (°C)	37	38	39	40	41	42	43
RKO	IC50 (µM)	2627	2627	2627	2627	3212	3212	3212
	TER	1.0	1.0	1.0	0.8	0.8	0.8	0.8
HCT116	IC50 (µM)	3183	2850	2666	2850	3183	2483	2000
	TER	1.0	1.1	1.2	1.1	1.0	1.3	1.6
MDST8	IC50 (µM)	130	175	200	130	157	150	130
	TER	1.0	0.8	0.7	1.0	0.8	0.9	1.0
COLO320	IC50 (µM)	250	300	300	268	211	250	227
	TER	1.0	1.0	1.0	1.0	1.2	1.0	1.1
HUTU80	IC50 (µM)	600	600	600	600	584	523	600
	TER	1.0	1.0	1.0	1.0	1.0	1.1	1.0

Supplementary Figure 5

A

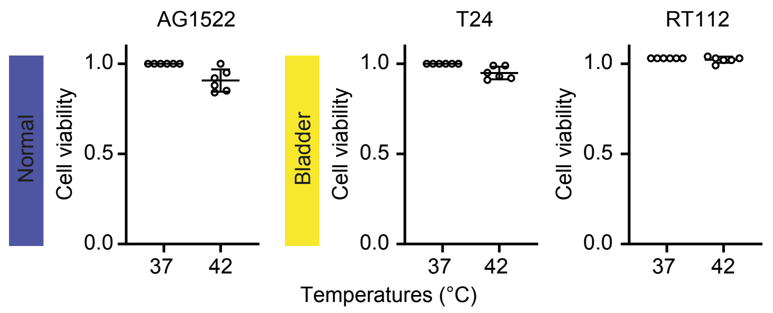

B

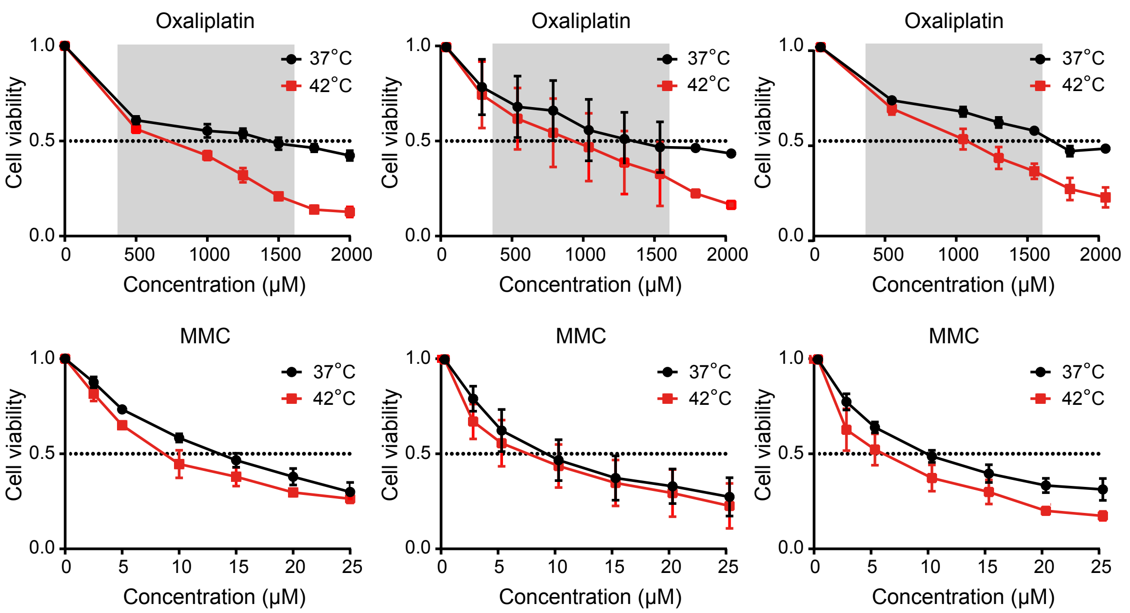

C

Oxaliplatin		37°C	42°C
AG1522	IC50 (μM)	1427	729
	TER	1.0	2.0
T24	IC50 (μM)	1341	908
	TER	1.0	1.5
RT112	IC50 (μM)	1630	1041
	TER	1.0	1.6

MMC		37°C	42°C
AG1522	IC50 (μM)	13.6	8.7
	TER	1.0	1.6
T24	IC50 (μM)	9.2	7.3
	TER	1.0	1.3
RT112	IC50 (μM)	9.8	5.6
	TER	1.0	1.8

D

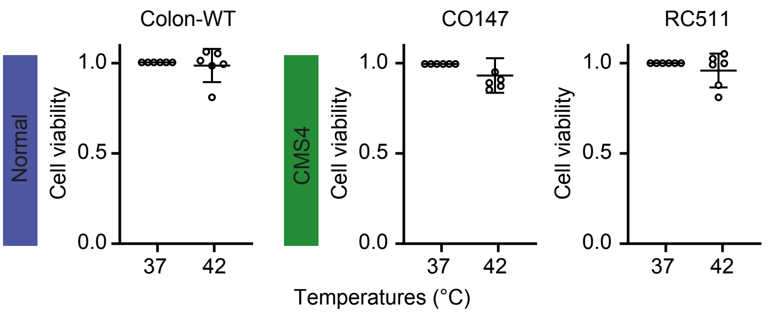

E

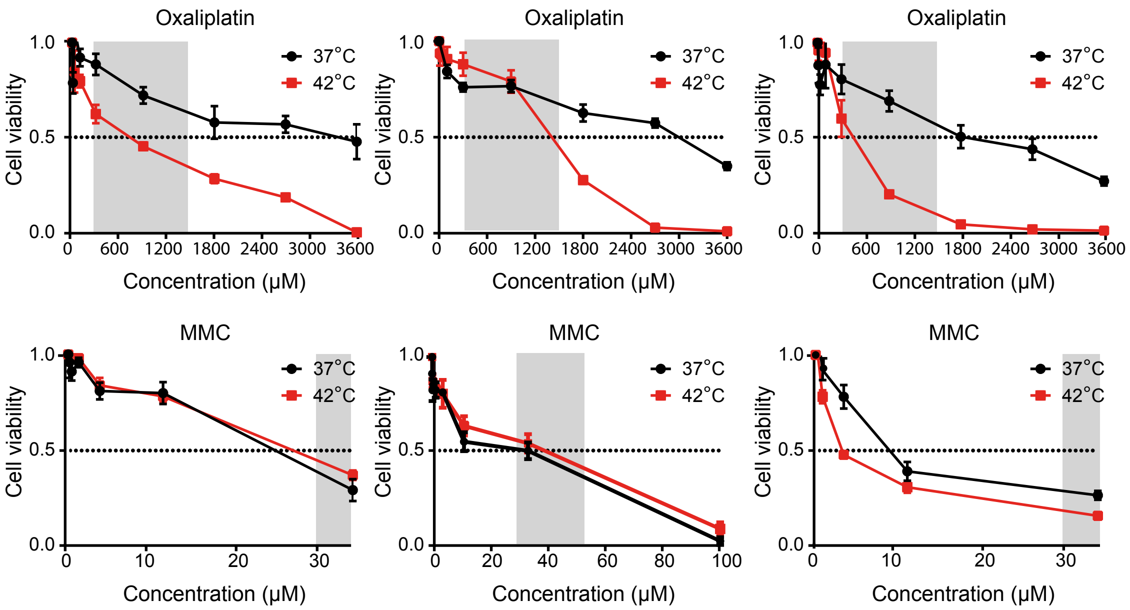

F

Oxaliplatin		37°C	42°C
Colon-WT	IC50 (μM)	3360	724
	TER	1.0	4.6
CO147	IC50 (μM)	2960	1386
	TER	1.0	2.1
RC511	IC50 (μM)	1844	434
	TER	1.0	4.2

MMC		37°C	42°C
Colon-WT	IC50 (μM)	24	26
	TER	1.0	0.9
CO147	IC50 (μM)	35	40
	TER	1.0	0.9
RC511	IC50 (μM)	8.9	3.8
	TER	1.0	2.3

Supplementary Figure 6

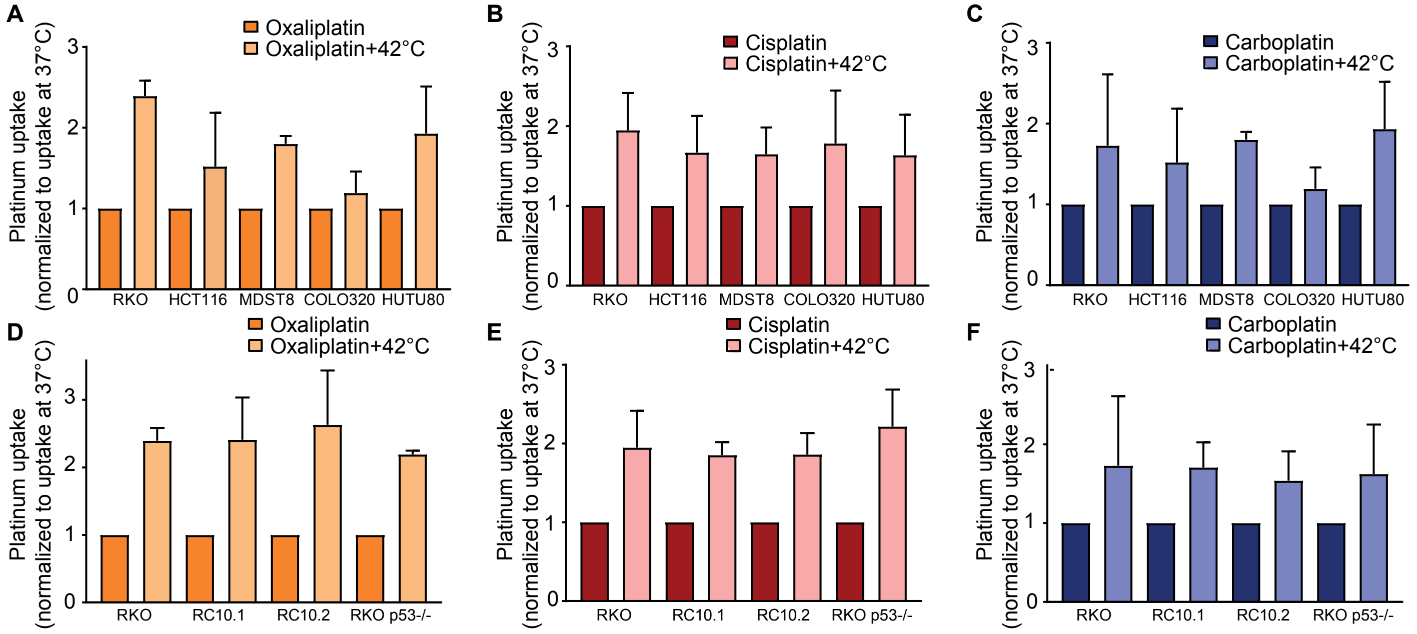

G

S6 A-C	Samples	p-value
RKO	Oxa vs. Oxa+42°C	0.1
	Cis vs. Cis+42°C	0.1
	Car vs. Car+42°C	0.7
HCT116	Oxa vs. Oxa+42°C	0.7
	Cis vs. Cis+42°C	0.1
	Car vs. Car+42°C	0.7
MDST8	Oxa vs. Oxa+42°C	0.1
	Cis vs. Cis+42°C	0.1
	Car vs. Car+42°C	0.1
COLO320	Oxa vs. Oxa+42°C	0.1
	Cis vs. Cis+42°C	0.1
	Car vs. Car+42°C	0.1
HUTU80	Oxa vs. Oxa+42°C	0.1
	Cis vs. Cis+42°C	0.1
	Car vs. Car+42°C	0.1

H

S6 D-F	Samples	p-value
RKO	Oxa vs. Oxa+42°C	0.1
	Cis vs. Cis+42°C	0.1
	Car vs. Car+42°C	0.7
RC10.1	Oxa vs. Oxa+42°C	0.1
	Cis vs. Cis+42°C	0.1
	Car vs. Car+42°C	0.1
RC10.2	Oxa vs. Oxa+42°C	0.1
	Cis vs. Cis+42°C	0.4
	Car vs. Car+42°C	0.1
RKO p53-/-	Oxa vs. Oxa+42°C	0.1
	Cis vs. Cis+42°C	0.1
	Car vs. Car+42°C	0.1

Supplementary Figure 7

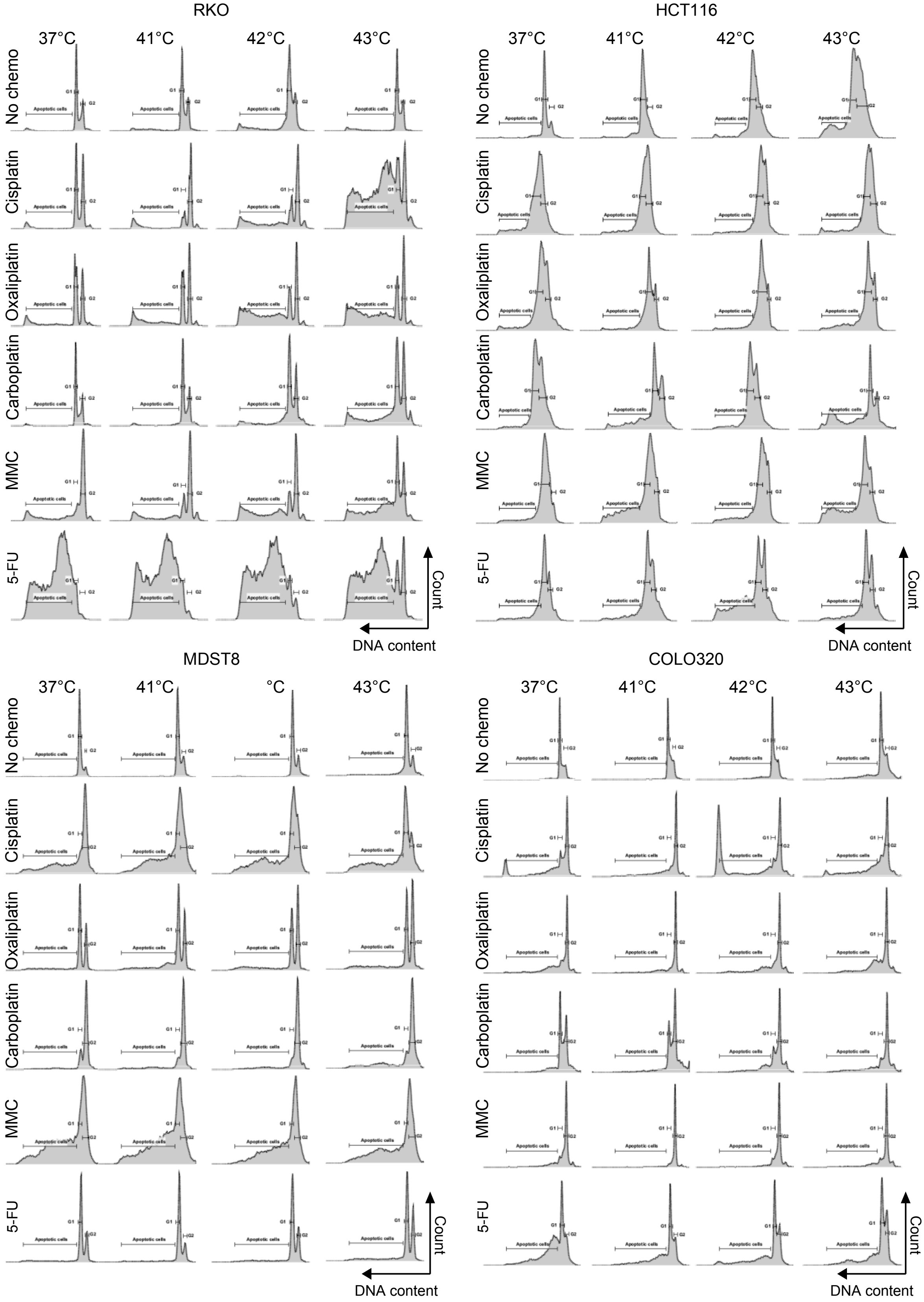

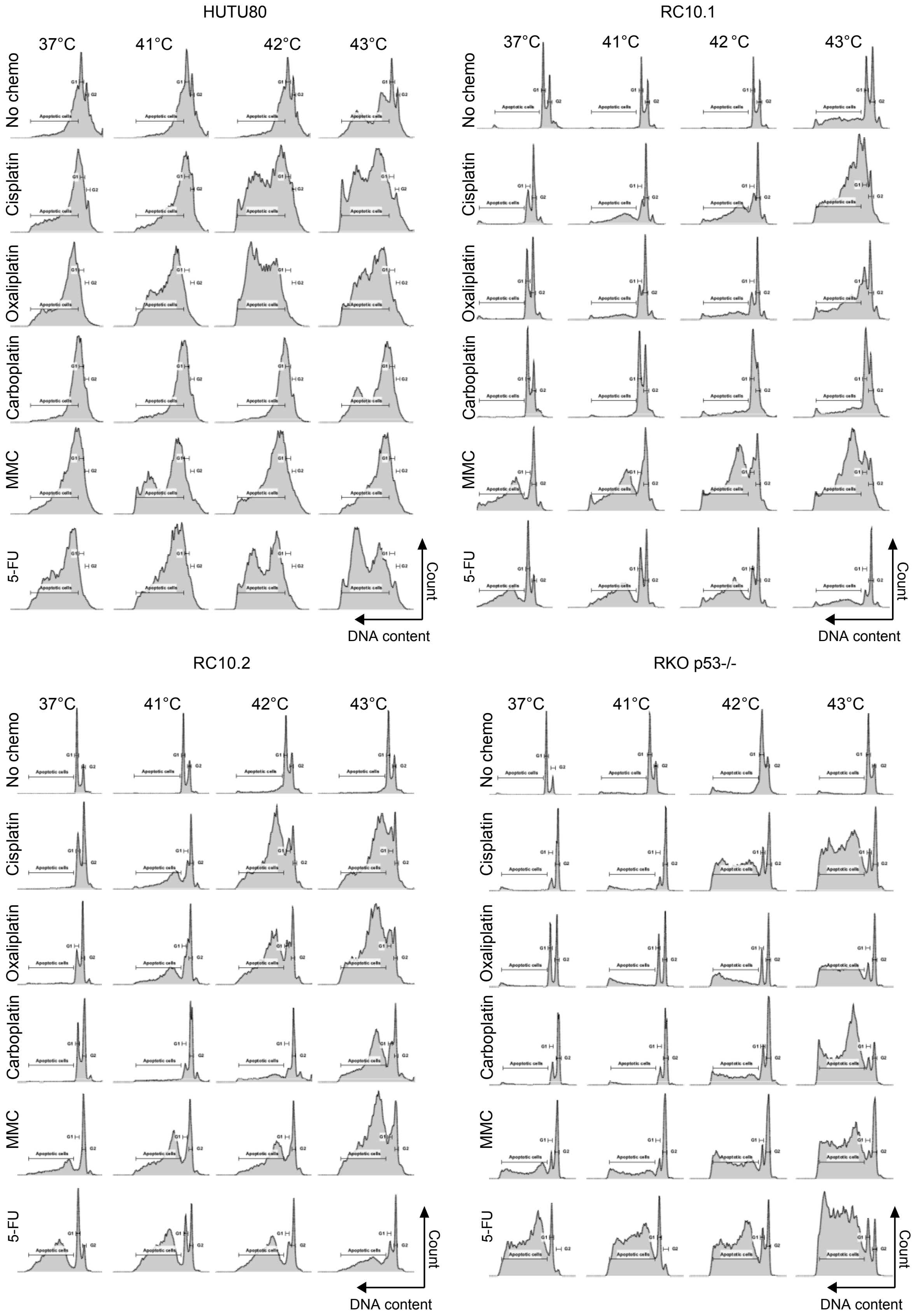
HUTU80
RC10.1
RC10.2
RKO p53-/-
37°C
41°C
42°C
43°C
No chemo
Cisplatin
Oxaliplatin
Carboplatin
MMC
5-FU
Apoptotic cells
G1
G2
Count
DNA content

Supplementary Figure 8

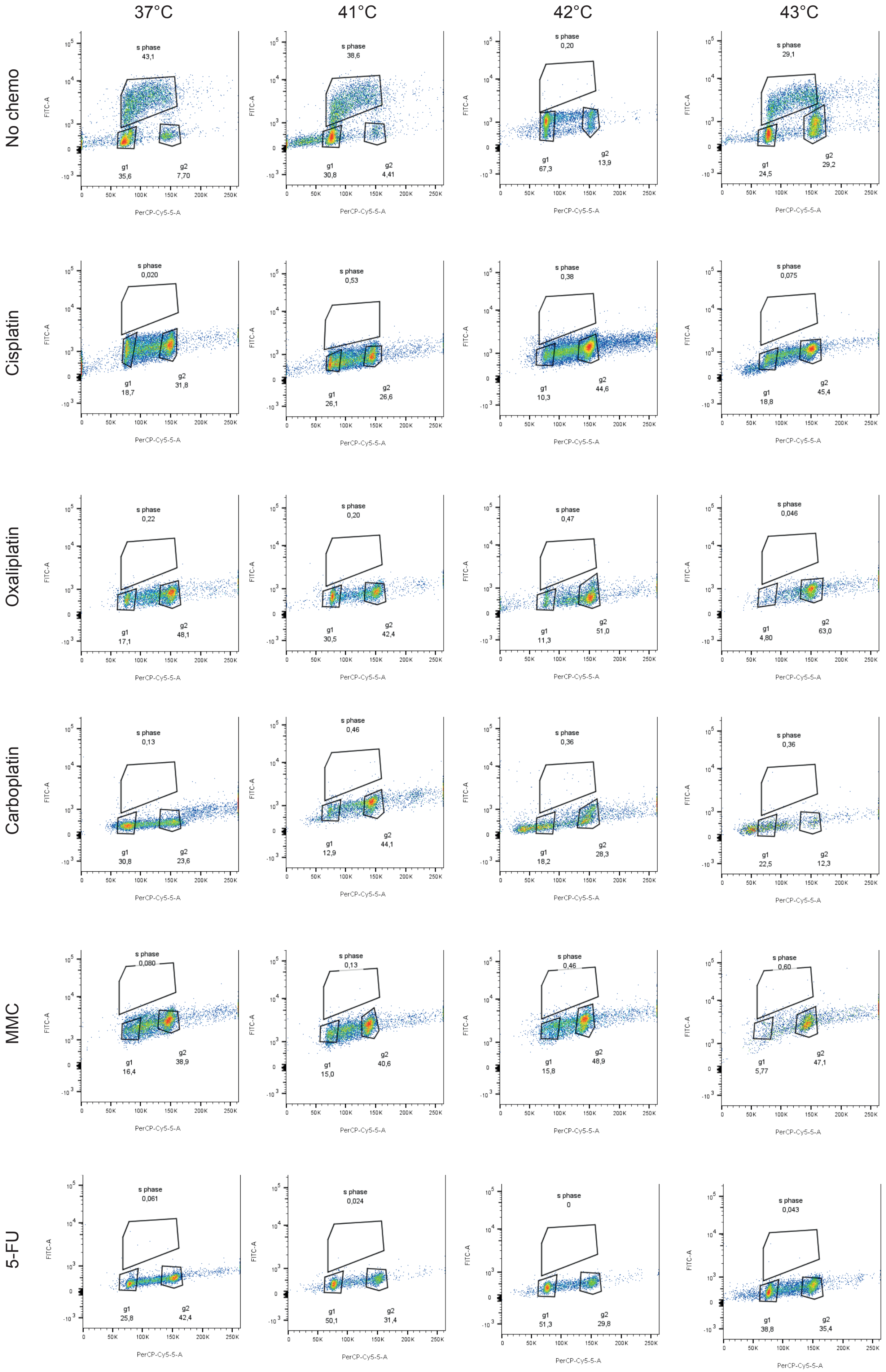

Supplementary Figure 9

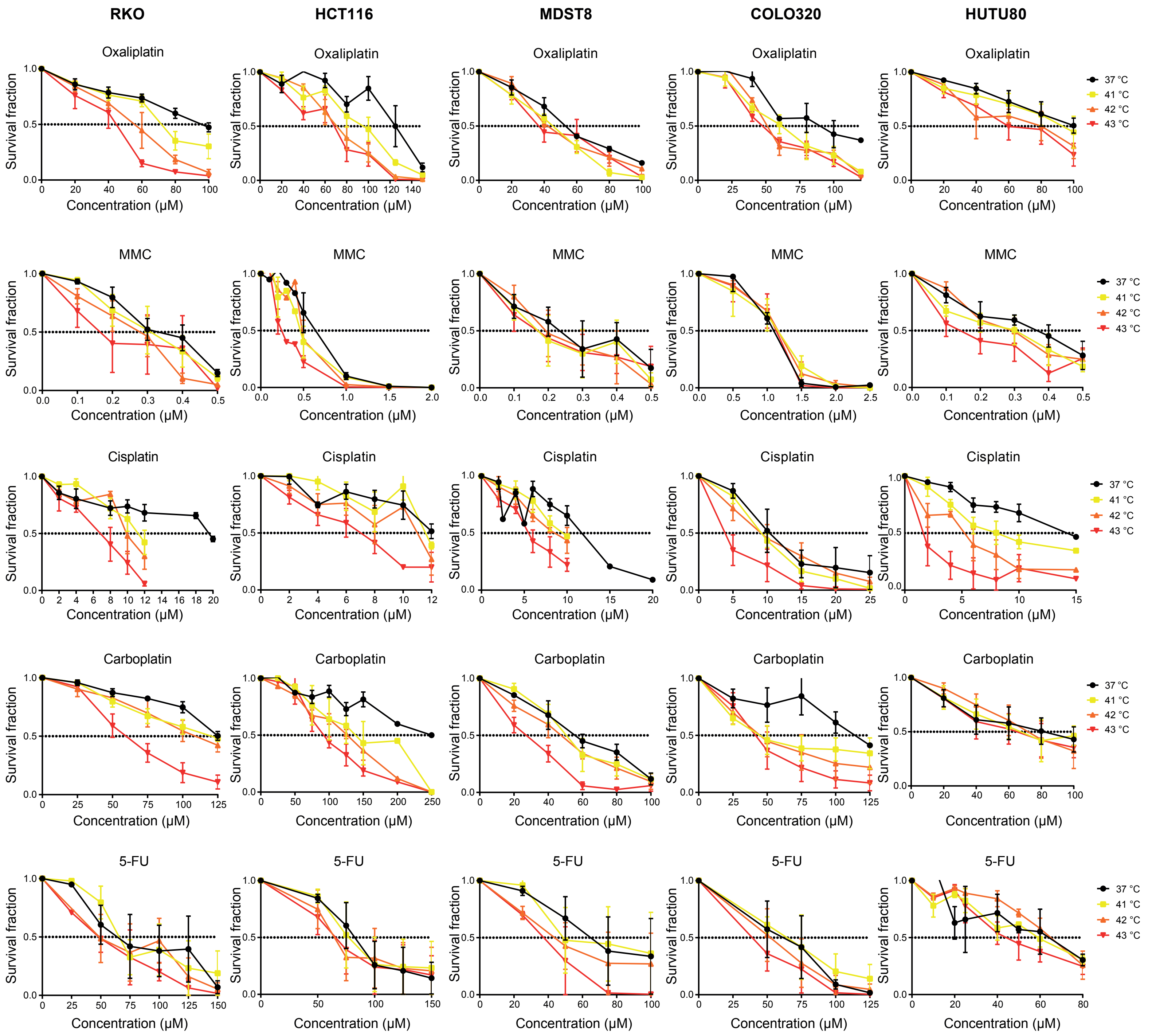

Supplementary Figure 10

Oxaliplatin	Temperature (°C)	37	41	42	43
RKO	IC50 (μM)	95.3	71.4	55.5	44.8
	TER	1.0	1.7	1.3	2.1
HCT116	IC50 (μM)	125	95	71	68
	TER	1.0	1.3	1.8	1.8
MDST8	IC50 (μM)	52.8	45.4	41.3	36.4
	TER	1.0	1.2	1.3	1.4
COLO320	IC50 (μM)	90	62	51	47
	TER	1.0	1.5	1.8	1.9
HUTU80	IC50 (μM)	100	93	80	60
	TER	1.0	1.1	1.3	1.7

MMC	Temperature (°C)	37	41	42	43
RKO	IC50 (μM)	0.33	0.31	0.28	0.16
	TER	1.0	1.1	1.2	2.1
HCT116	IC50 (μM)	0.64	0.46	0.48	0.24
	TER	1.0	1.4	1.3	2.7
MDST8	IC50 (μM)	0.23	0.17	0.19	0.17
	TER	1.0	1.4	1.2	1.4
COLO320	IC50 (μM)	1.1	1.2	1.1	1.2
	TER	1.0	1.0	1.0	1.0
HUTU80	IC50 (μM)	0.36	0.3	0.3	0.14
	TER	1.0	1.2	1.2	2.6

Cisplatin	Temperature (°C)	37	41	42	43
RKO	IC50 (μM)	19.5	11.2	9.9	6.8
	TER	1.0	1.7	2.0	2.9
HCT116	IC50 (μM)	12	11.5	11	7.0
	TER	1.0	1.0	1.1	1.7
MDST8	IC50 (μM)	11.5	9.4	8.6	5.5
	TER	1.0	1.6	1.7	2.1
COLO320	IC50 (μM)	10.4	9.0	9.0	3.8
	TER	1.0	1.2	1.2	2.7
HUTU80	IC50 (μM)	14.2	8.0	5.2	1.5
	TER	1.0	1.8	2.7	9.5

Carboplatin	Temperature (°C)	37	41	42	43
RKO	IC50 (μM)	125	120	109	59.7
	TER	1.0	1.0	1.2	2.1
HCT116	IC50 (μM)	250	138	125	92
	TER	1.0	1.8	2.0	2.7
MDST8	IC50 (μM)	55.3	50.6	47.3	26.8
	TER	1.0	1.1	1.2	2.1
COLO320	IC50 (μM)	114	44	44	41
	TER	1.0	2.6	2.6	2.8
HUTU80	IC50 (μM)	80	67	71	65
	TER	1.0	1.2	1.1	1.2

5-FU	Temperature (°C)	37	41	42	43
RKO	IC50 (μM)	64.2	65.7	48.7	48.7
	TER	1.0	1.0	1.3	1.3
HCT116	IC50 (μM)	83	77	65	65
	TER	1.0	1.1	1.3	1.3
MDST8	IC50 (μM)	65	49	44	37
	TER	1.0	1.3	1.5	1.8
COLO320	IC50 (μM)	62	64	52	39
	TER	1.0	1.0	1.2	1.6
HUTU80	IC50 (μM)	65	60	65	44
	TER	1.0	1.1	1.0	1.5

Supplementary Figure 11

Figure 2C	Samples	p-value
RKO	Oxa vs. Oxa+42°C	0.200
HCT116	Oxa vs. Oxa+42°C	0.700
MDST8	Oxa vs. Oxa+42°C	0.100
COLO320	Oxa vs. Oxa+42°C	0.200
HUTU80	Oxa vs. Oxa+42°C	0.100

Figure 2I	Samples	p-value
RKO	Oxa vs. Oxa+42°C	0.029
HCT116	Oxa vs. Oxa+42°C	0.029
MDST8	Oxa vs. Oxa+42°C	0.314
COLO320	Oxa vs. Oxa+42°C	0.029
HUTU80	Oxa vs. Oxa+42°C	0.114

Figure 2J	Samples	p-value
RKO	MMC vs. MMC+42°C	0.333
HCT116	MMC vs. MMC+42°C	0.400
MDST8	MMC vs. MMC+42°C	0.700
COLO320	MMC vs. MMC+42°C	0.886
HUTU80	MMC vs. MMC+42°C	0.999

Figure 2F	Samples	p-value
RKO	Control vs. 42°C	0.029
	Oxa vs. Oxa+42°C	0.100
	MMC vs. MMC+42°C	0.400
HCT116	Control vs. 42°C	0.700
	Oxa vs. Oxa+42°C	0.700
	MMC vs. MMC+42°C	0.400
MDST8	Control vs. 42°C	0.999
	Oxa vs. Oxa+42°C	0.400
	MMC vs. MMC+42°C	0.700
COLO320	Control vs. 42°C	0.300
	Oxa vs. Oxa+42°C	0.400
	MMC vs. MMC+42°C	0.999
HUTU80	Control vs. 42°C	0.800
	Oxa vs. Oxa+42°C	0.700
	MMC vs. MMC+42°C	0.999

Figure 3C	Samples	p-value
RKO	Cis vs. Cis+42°C	0.400
HCT116	Cis vs. Cis+42°C	0.400
MDST8	Cis vs. Cis+42°C	0.200
COLO320	Cis vs. Cis+42°C	0.400
HUTU80	Cis vs. Cis+42°C	0.400

Figure 3D	Samples	p-value
RKO	Car vs. Car+42°C	0.999
HCT116	Car vs. Car+42°C	0.999
MDST8	Car vs. Car+42°C	0.100
COLO320	Car vs. Car+42°C	0.700
HUTU80	Car vs. Car+42°C	0.100

Figure 3H	Samples	p-value
RKO	Cis vs. Cis+42°C	0.029
HCT116	Cis vs. Cis+42°C	0.486
MDST8	Cis vs. Cis+42°C	0.114
COLO320	Cis vs. Cis+42°C	0.999
HUTU80	Cis vs. Cis+42°C	0.029

Figure 3J	Samples	p-value
RKO	Car vs. Car+42°C	0.029
HCT116	Car vs. Car+42°C	0.029
MDST8	Car vs. Car+42°C	0.999
COLO320	Car vs. Car+42°C	0.057
HUTU80	Car vs. Car+42°C	0.686

Figure 3E	Samples	p-value
RKO	Control vs. 42°C	0.029
	Cis vs. Cis+42°C	0.100
	Car vs. Car+42°C	0.400
HCT116	Control vs. 42°C	0.700
	Cis vs. Cis+42°C	0.400
	Car vs. Car+42°C	0.400
MDST8	Control vs. 42°C	0.999
	Cis vs. Cis+42°C	0.700
	Car vs. Car+42°C	0.400
COLO320	Control vs. 42°C	0.300
	Cis vs. Cis+42°C	0.700
	Car vs. Car+42°C	0.700
HUTU80	Control vs. 42°C	0.800
	Cis vs. Cis+42°C	0.999
	Car vs. Car+42°C	0.700

Figure 4B	Samples	p-value
RKO	5-FU vs. 5-FU+42°C	0.999
HCT116	5-FU vs. 5-FU+42°C	0.400
MDST8	5-FU vs. 5-FU+42°C	0.999
COLO320	5-FU vs. 5-FU+42°C	0.999
HUTU80	5-FU vs. 5-FU+42°C	0.400

Figure 4E	Samples	p-value
RKO	5-FU vs. 5-FU+42°C	0.999
HCT116	5-FU vs. 5-FU+42°C	0.486
MDST8	5-FU vs. 5-FU+42°C	0.400
COLO320	5-FU vs. 5-FU+42°C	0.800
HUTU80	5-FU vs. 5-FU+42°C	0.686

Figure 5D	Samples	p-value
RKO	Oxa vs. Oxa+42°C	0.200
RC10.1	Oxa vs. Oxa+42°C	0.100
RC10.2	Oxa vs. Oxa+42°C	0.100
RKO p53-/-	Oxa vs. Oxa+42°C	0.200
RKO	Cis vs. Cis+42°C	0.400
RC10.1	Cis vs. Cis+42°C	0.400
RC10.2	Cis vs. Cis+42°C	0.400
RKO p53-/-	Cis vs. Cis+42°C	0.200
RKO	Car vs. Car+42°C	0.999
RC10.1	Car vs. Car+42°C	0.400
RC10.2	Car vs. Car+42°C	0.400
RKO p53-/-	Car vs. Car+42°C	0.400

Figure 5F	Samples	p-value
RKO	Oxa vs. Oxa+42°C	0.029
RC10.1	Oxa vs. Oxa+42°C	0.200
RC10.2	Oxa vs. Oxa+42°C	0.029
RKO p53-/-	Oxa vs. Oxa+42°C	0.086

Figure 5G	Samples	p-value
RKO	MMC vs. MMC+42°C	0.333
RC10.1	MMC vs. MMC+42°C	0.999
RC10.2	MMC vs. MMC+42°C	0.999
RKO p53-/-	MMC vs. MMC+42°C	0.999

Figure 5H	Samples	p-value
RKO	Cis vs. Cis+42°C	0.029
RC10.1	Cis vs. Cis+42°C	0.029
RC10.2	Cis vs. Cis+42°C	0.029
RKO p53-/-	Cis vs. Cis+42°C	0.114

Figure 5I	Samples	p-value
RKO	Car vs. Car+42°C	0.029
RC10.1	Car vs. Car+42°C	0.171
RC10.2	Car vs. Car+42°C	0.029
RKO p53-/-	Car vs. Car+42°C	0.057

Figure 5J	Samples	p-value
RKO	5-FU vs. 5-FU+42°C	0.999
RC10.1	5-FU vs. 5-FU+42°C	0.800
RC10.2	5-FU vs. 5-FU+42°C	0.200
RKO p53-/-	5-FU vs. 5-FU+42°C	0.999
